# Supplementary material for: Increasingly efficient chromatin binding of cohesin and CTCF supports chromatin architecture formation during zebrafish embryogenesis
Source: Nat Commun. 2025 Feb 21;16:1833. doi: 10.1038/s41467-025-56889-5 (PMC11842872; doi:10.1038/s41467-025-56889-5)
Supplement: Supplementary file 1 — Supplementary Information [file 41467_2025_56889_MOESM1_ESM.pdf]

Supplementary Information

Increasingly efficient chromatin binding of cohesin and CTCF supports chromatin architecture formation during zebrafish embryogenesis

Jonas Coßmann[1,4], Pavel I. Kos[2,5], Vassiliki Varamogianni-Mamatsi[3], Devin S. Assenheimer[1,4], Tobias A. Bischof[1,4], Timo Kuhn[1], Thomas Vomhof[1], Argyris Papantonis[3], Luca Giorgetti[2], J. Christof M. Gebhardt[#,1,5]

[1]Institute of Biophysics, Ulm University, Ulm, Germany
[2]Friedrich Miescher Institute for Biomedical Research, Basel, Switzerland
[3]Institute of Pathology, University Medical Center Göttingen, Göttingen, Germany
[4]present address: Institute of Experimental Physics and IQST, Ulm University, Ulm, Germany
[5]present address: Department of Biomedicine, University of Basel, Basel, Switzerland

[#]To whom correspondence should be addressed:
christof.gebhardt@uni-ulm.de

Contents

Supplementary Figures 2

Supplementary Tables 21

Supplementary Movie Legends 31

Supplementary Figures

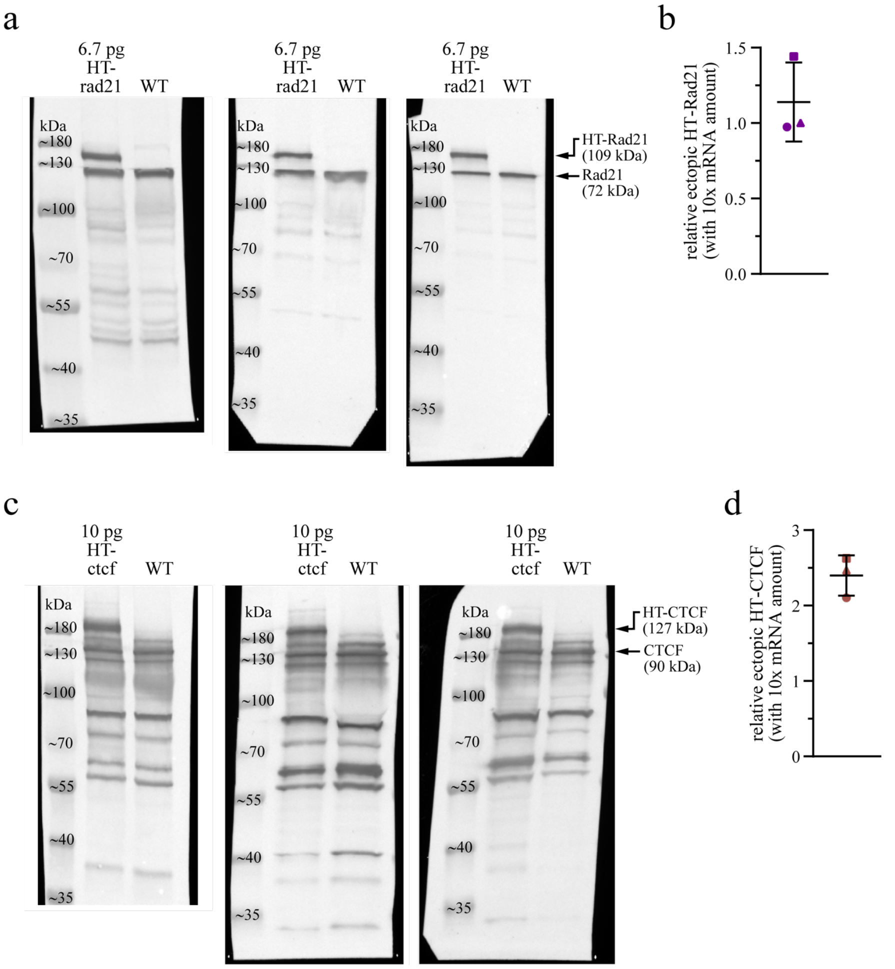

Supplementary Figure 1. Quantification of HT-Rad21 and HT-CTCF expression levels in shield-stage zebrafish embryos. a), c) Western blots (WB) of three independent biological replicates of uninjected embryos (WT) or embryos injected with 10-fold increased injection amounts compared to our single-molecule measurements to enhance band clarity (see Methods). **a)** WB with anti-Rad21 antibody of embryos injected with 6.7 pg HT-rad21 mRNA or uninjected embryos (WT). **b)** Quantification of a) comparing the HT-Rad21 band to the endogenous Rad21 band. Single-molecule injections were performed with 10-fold decreased mRNA amount yielding (11 ± 3)% of ectopic HT-Rad21 expression. **c)** WB with anti-CTCF antibody of embryos injected 10 pg HT-ctcf mRNA or uninjected embryos (WT). **d)** Quantification of c) comparing the HT-CTCF band to the endogenous CTCF band. Single-molecule injections were performed with 10-fold decreased mRNA amount yielding (24 ± 3)% of ectopic HT-CTCF expression. Lines represent mean values ± s.d. Source data are provided as a Source Data file for Supplementary Fig. 2b, d.

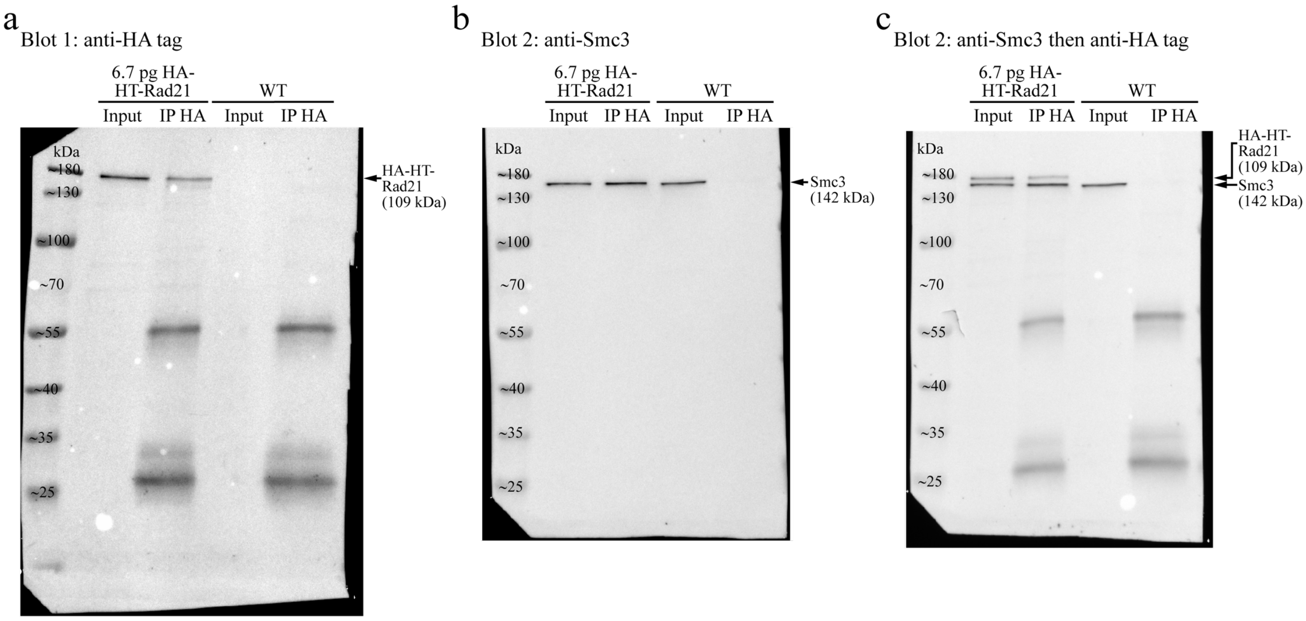

Supplementary Figure 2. Co-immunoprecipitation (Co-IP) of Smc3 with HA-HT-Rad21 in zebrafish embryos. SDS-page gel stained with **a)** anti-HA tag antibody (Blot 1) and **b)** anti-Smc3 antibody (Blot 2). **c)** Blot 2 from panel b) subsequently stained with anti-HA tag antibody. Gels were loaded with: lysate of embryos injected with mRNA encoding for HA-tagged HT-Rad21 (lane 1, Input), immunoprecipitation (IP) using an anti-HA tag antibody (lane 2, IP HA), lysate of uninjected embryos (lane 3, WT, Input), IP using an anti-HA tag antibody (lane 4, IP HA). Endogenous Smc3 was detected in both the HA-HT-rad21 injected and uninjected WT input samples. Smc3 was also detected in the IP HA samples from injected embryos. HA-HT-Rad21 protein was only detected in embryos injected with the HA-HT-rad21 mRNA and not in the WT control.

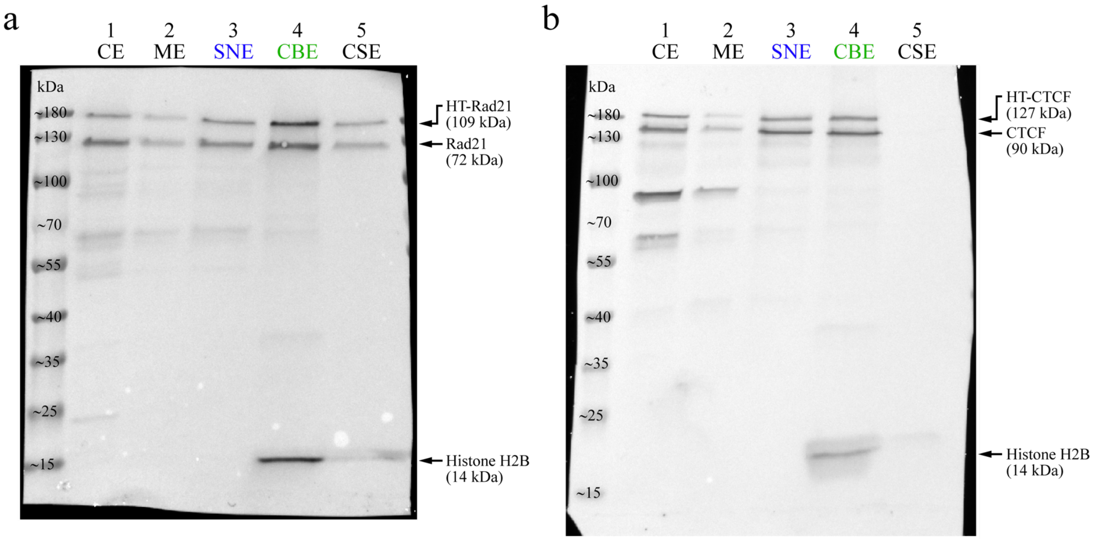

Supplementary Figure 3. Subcellular protein fractionation of HT-tagged wild-type proteins from lysates of shield-stage zebrafish embryos. a)-b) Western blots of subcellular protein fractionated shield-stage embryos injected in the 1-cell stage with **a)** 6.7 pg HT-rad21 mRNA or **b)** 10 pg HT-ctcf mRNA. Blots were probed with anti-Rad21 and anti-Histone H2B antibody (a)), or anti-CTCF and anti-Histone H2B antibody (b)). Embryos were injected with a 10-fold injection amount compared to our single-molecule measurements to enhance band clarity (see Methods). Lanes include extracts of: 1-CE: Cytoplasmic, 2-ME: Membrane, 3-SNE: Soluble nuclear, 4-CBE: Chromatin bound, 5-CSE: Cytoskeletal extracts.

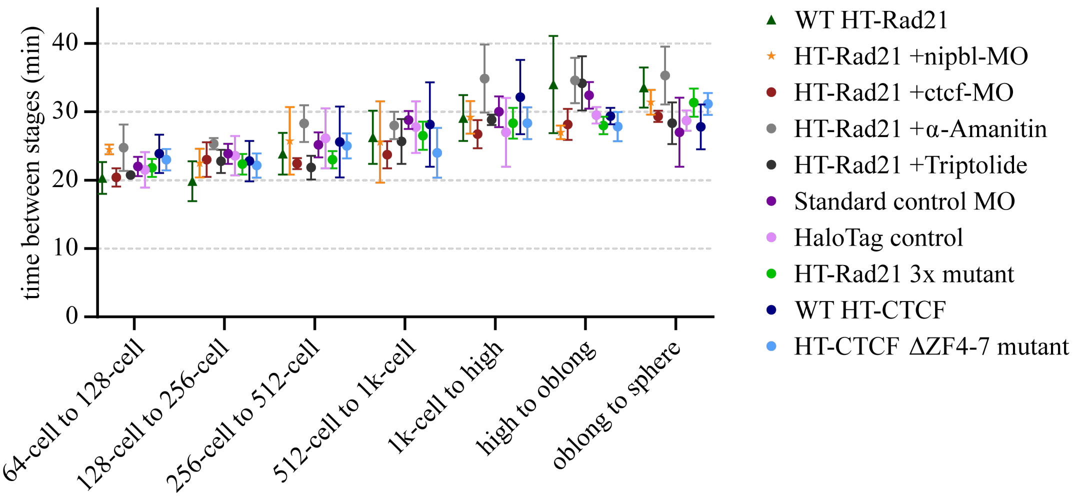

Supplementary Figure 4. Time intervals between developmental stages of early zebrafish embryos injected with different mRNAs or treatments. Time between developmental stages was determined by comparing time-stamps of consecutive raw movies for each embryo (see Methods). Data represent mean values ± s.d. Source data are provided as a Source Data file.

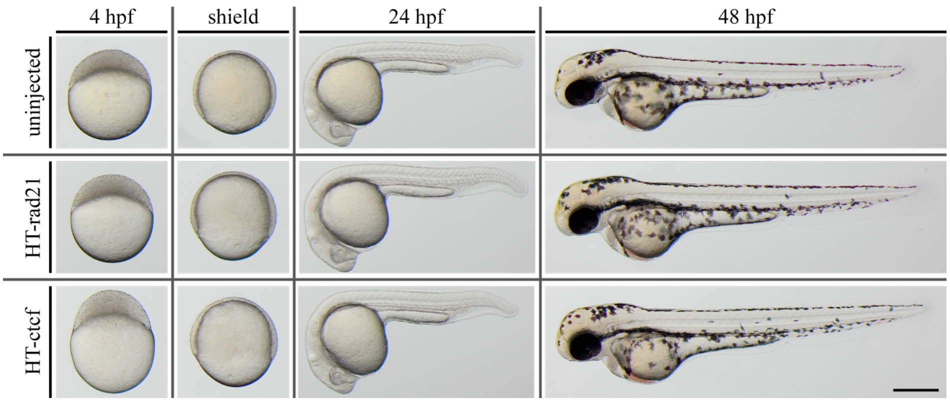

Supplementary Figure 5. Development for HT-rad21 and HT-ctcf injected zebrafish embryos. Lateral views of whole embryos either uninjected or injected with HT-rad21 mRNA or HT-ctcf mRNA (see Methods). Stages are provided according to Kimmel et al., 1995. Scale bar is 500 µm and applies to all images.

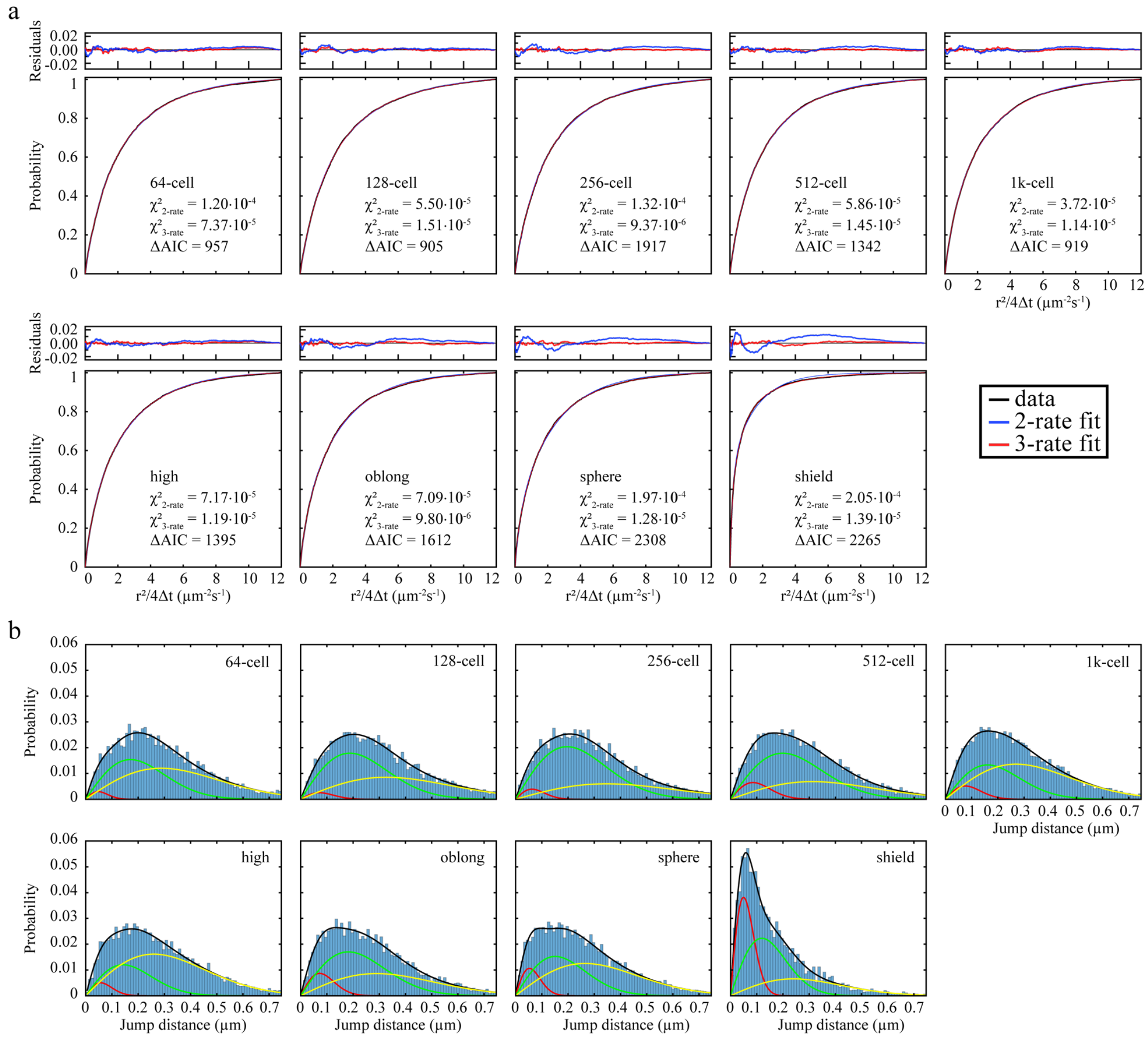

Supplementary Figure 6. Analysis of jump distance distributions of HT-Rad21 mobility data. **a**) Cumulative distributions of jump distances (black line) taken from 11.7 ms continuous movies with fits of a 2-component (blue) and 3-component (red) diffusion model at indicated developmental stages. Values give the reduced χ^2 and Akaike Information Criterion (AIC), evidencing a preferable 3-component fit. **b**) Distributions of jump distances with a three-component diffusion model (black) and every single component (red, green, yellow) at indicated developmental stages.

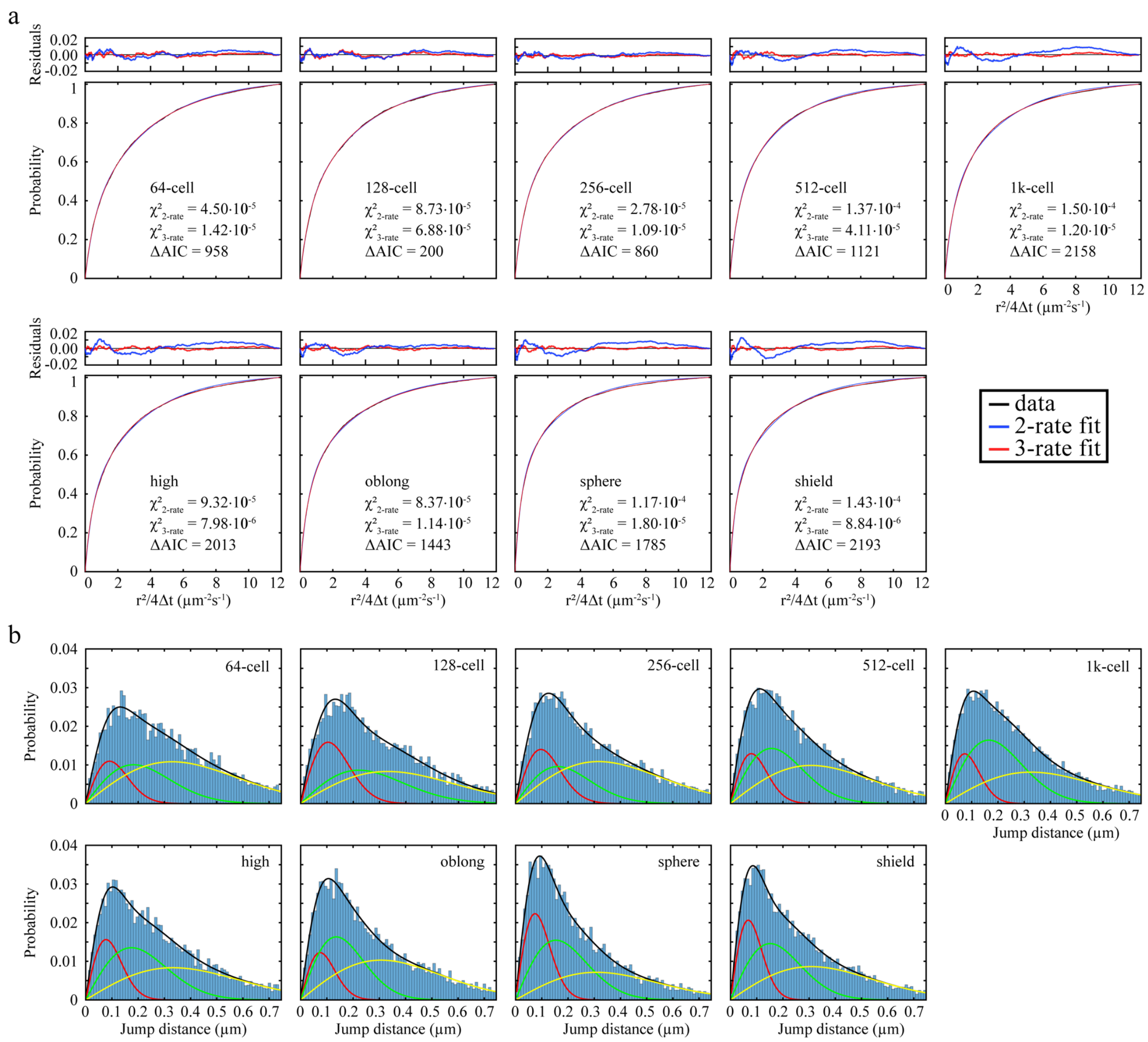

Supplementary Figure 7. Analysis of jump distance distributions of HT-CTCF mobility data. **a**) Cumulative distributions of jump distances (black line) taken from 11.7 ms continuous movies with fits of a 2-component (blue) and 3-component (red) diffusion model at indicated developmental stages. Values give the reduced χ^2 and Akaike Information Criterion (AIC), evidencing a preferable 3-component fit. **b**) Distribution of jump distances with a three-component diffusion model (black) and every single component (red, green, yellow) at indicated developmental stages.

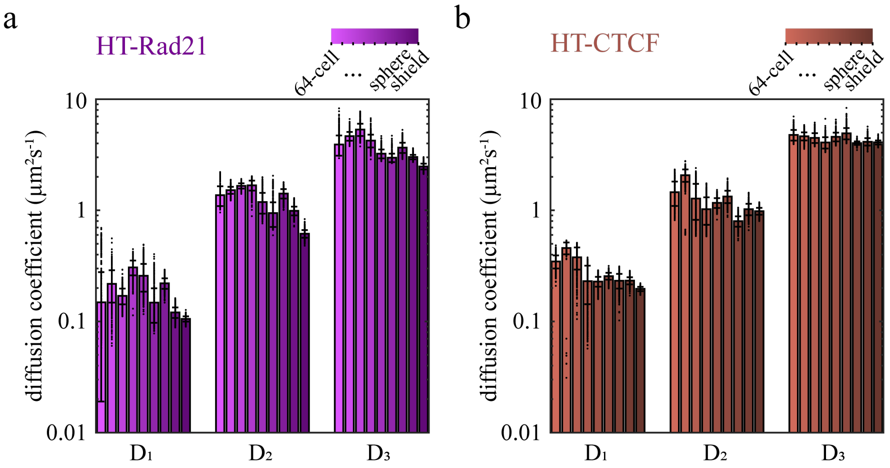

Supplementary Figure 8. Mobility of HT-Rad21 and HT-CTCF during zebrafish development. Diffusion coefficients of **a)** HT-Rad21 and **b)** HT-CTCF obtained from a three-component diffusion model fitted to jump distance distributions. Colors indicate stages of development (64-, 128-, 256-, 512-, 1k-cell, high, oblong, sphere, shield). Bars represent mean values ± s.d. from 500 resamplings using 80% of randomly selected jump distances. For amplitudes, see Fig. 1. Source data are provided as a Source Data file.

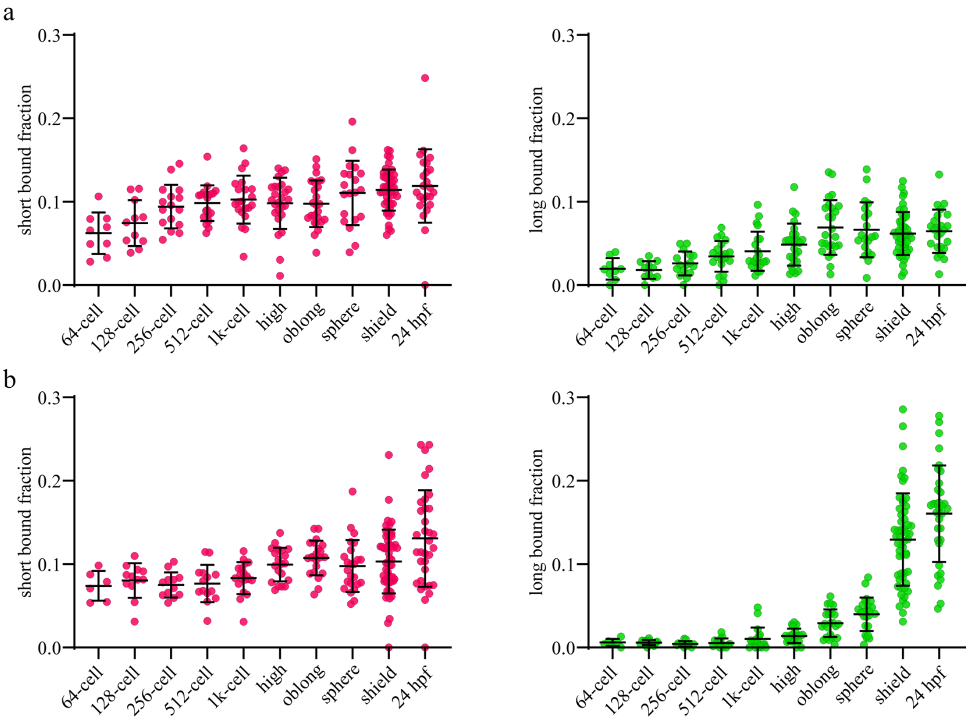

Supplementary Figure 9. Raw data plots for HT-CTCF and HT-Rad21. Raw data for Fig. 1g and h are shown for the fractions of short (left) and long (right) binding events of **a)** HT-CTCF and **b)** HT-Rad21. Data represent mean ± s.d. Source data are provided as a Source Data file for the original Fig. 1g, h.

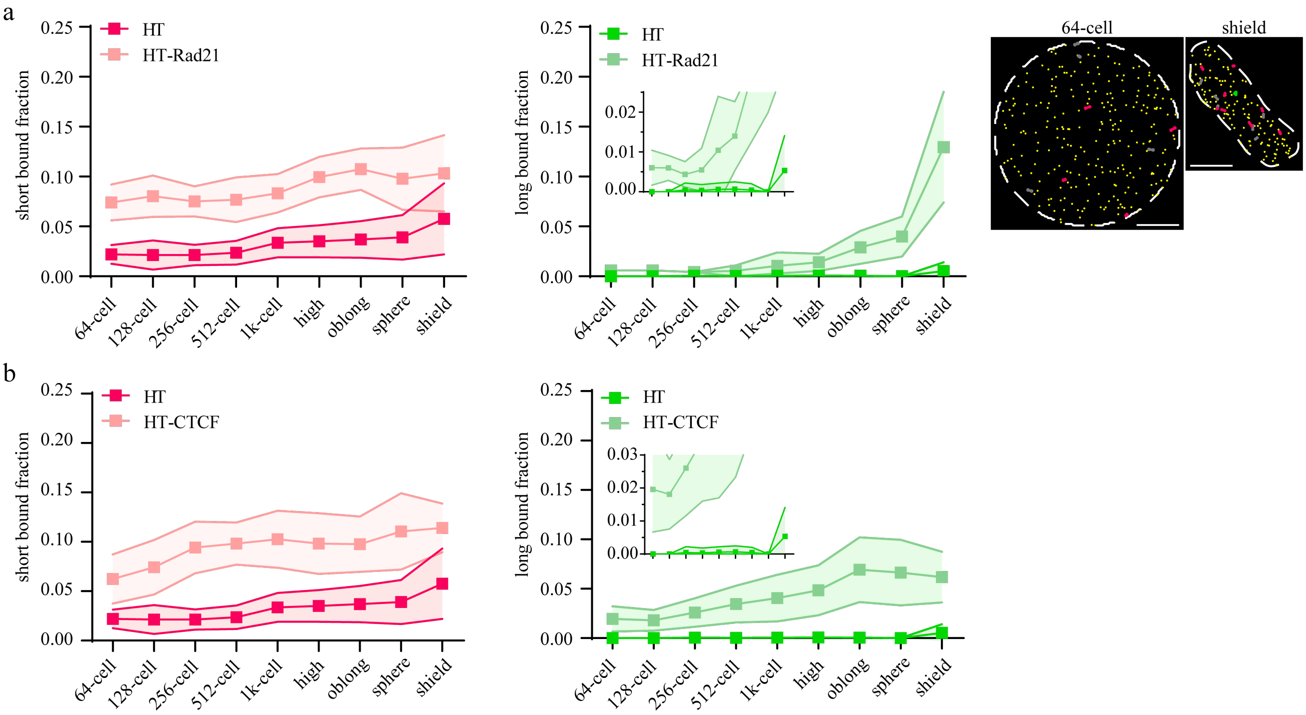

Supplementary Figure 10. HaloTag (HT) control recorded with interlaced time-lapse microscopy (ITM). **a), b)** Left: Fractions of short and long binding events of HT control overlaid with **a)** HT-Rad21 or **b)** HT-CTCF recorded with ITM illumination. **a)** Right: Example nuclei from ITM movies of zebrafish embryos injected with RNA encoding for HT control. Tracks are colored according to the binding classes given in Fig. 1f. Grey tracks survived only one long dark time. Scale bar: 5 µm. Data represent mean ± s.d. of movie-wise determined fractions. Insets show zooms in the respective graphs. Lines serve as guides to the eye. Statistics and p-values for HT control are provided in Supplementary Tables 17-18 and for HT-Rad21/HT-CTCF in Supplementary Tables 6-7. Raw dot plots for HT control are provided in Supplementary Fig. 11 and for HT-Rad21/HT-CTCF in Supplementary Fig. 9. Source data are provided as a Source Data file.

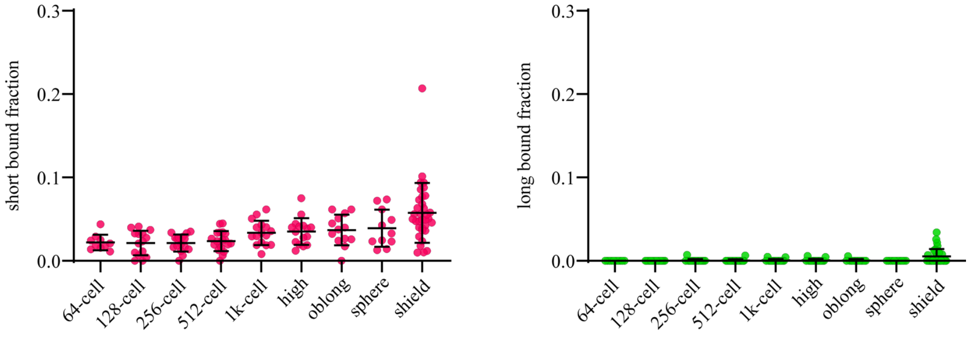

Supplementary Figure 11. Raw data plots for HaloTag (HT) control. Raw data are shown for the fractions of short (left) and long (right) binding events of the HT control recorded with interlaced time-lapse microscopy (ITM) illumination (compare Supplementary Fig. 10). Data represent mean ± s.d. Source data are provided as a Source Data file for the original Supplementary Fig. 10.

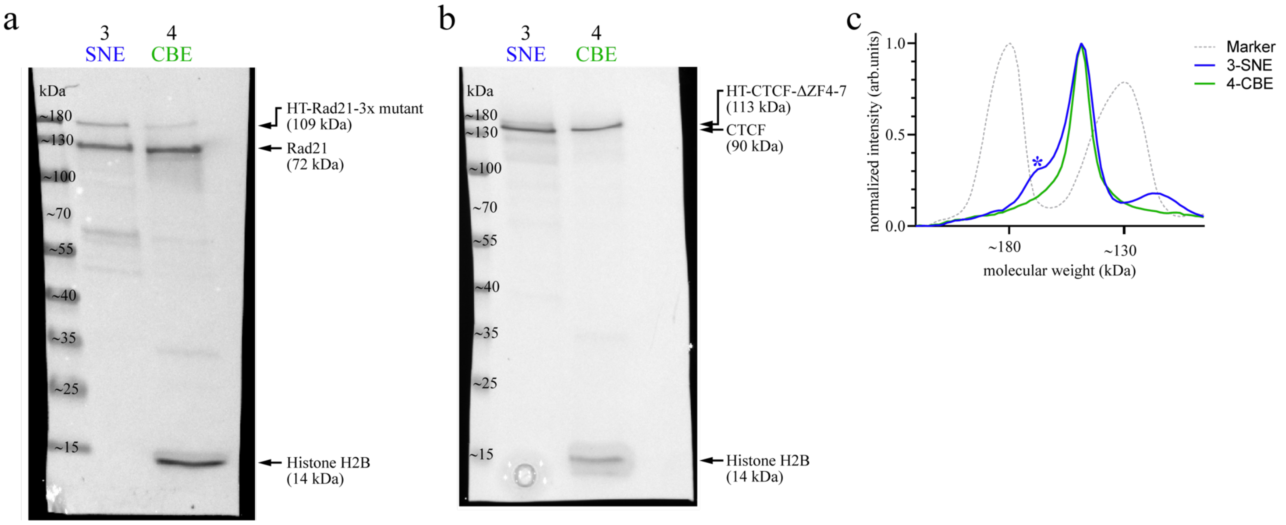

Supplementary Figure 12. Subcellular protein fractionation of HT-tagged mutant proteins from lysates of shield-stage zebrafish embryos. a)-b) Western blots of subcellular protein fractionated shield-stage embryos injected in the 1-cell stage with **a)** 6.7 pg HT-rad21-3x mutant mRNA or **b)** 10 pg HT-ctcf-ΔZF4-7 mutant mRNA. Blots were probed with anti-Rad21 and anti-Histone H2B antibody (a)), or anti-CTCF and anti-Histone H2B antibody (b)). **c)** Intensity plot for Western blot bands depicted in panel b) between ~180-130 kDa (refer to grey marker lane), quantified across a straight region of each band (see Methods). Asterisk denotes the intensity maximum corresponding to HT-CTCF-ΔZF4-7 protein. Embryos were injected with a 10-fold injection amount compared to our single-molecule measurements to enhance band clarity (see Methods). Lanes include extracts of: 1-CE: Cytoplasmic, 2-ME: Membrane, 3-SNE: Soluble nuclear, 4-CBE: Chromatin bound, 5-CSE: Cytoskeletal extracts.

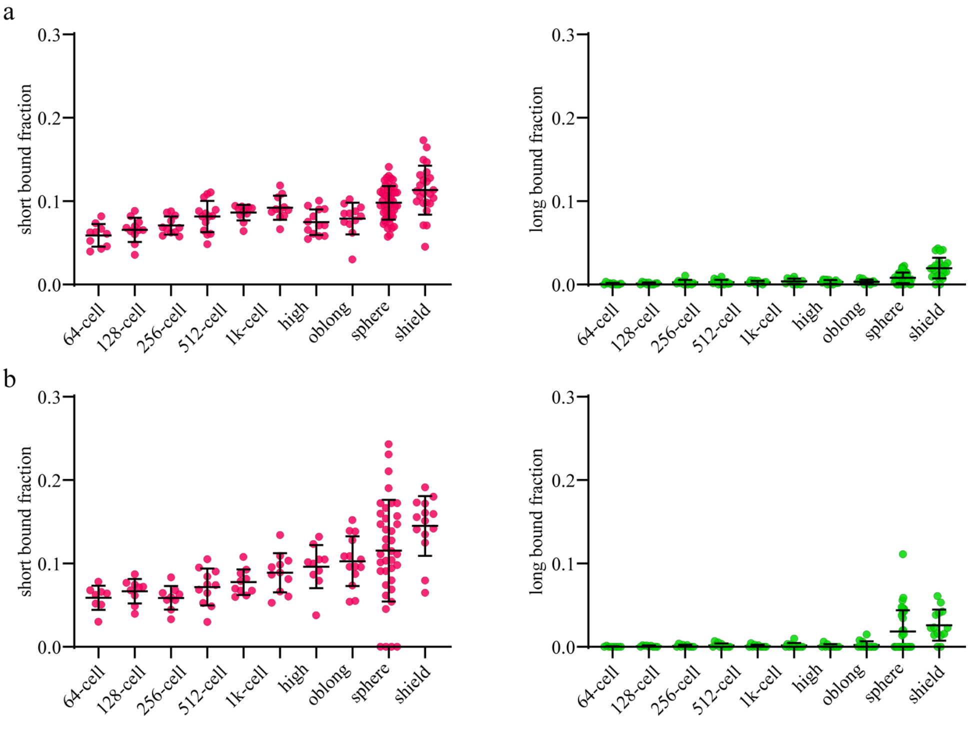

Supplementary Figure 13. Raw data plots for HT-CTCF-ΔZF4-7 and HT-Rad21-3x-mutant. Raw data for Fig. 1g and h are shown for the fractions of short (left) and long (right) binding events of **a)** HT-CTCF-ΔZF4-7 and **b)** HT-Rad21-3x-mutant recorded with interlaced time-lapse microscopy (ITM) illumination (compare Fig. 1g, h). Data represent mean ± s.d. Source data are provided as a Source Data file for the original Fig. 1g, h.

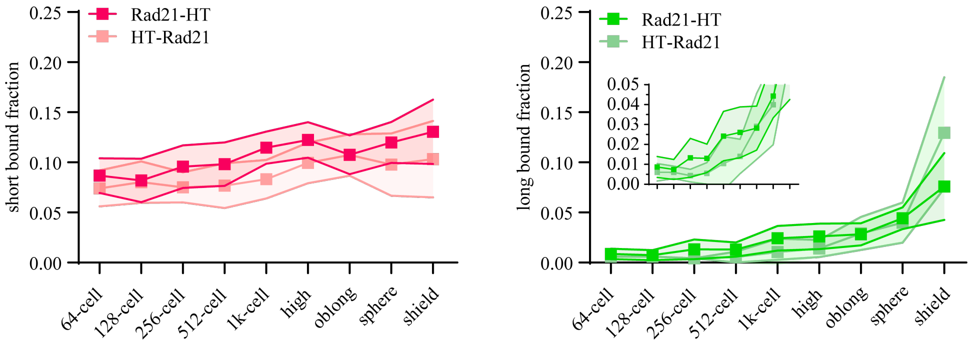

Supplementary Figure 14. Comparison of bound fractions of HT-Rad21 and Rad21-HT. Fractions of short and long binding events of Rad21-HT or HT-Rad21 obtained by ITM measurements. Data represent mean ± s.d. of movie-wise determined fractions. Inset shows zoom into the respective graph. Lines serve as guides to the eye. Statistics and p-values for Rad21-HT are provided in Supplementary Tables 19-20 and for HT-Rad21 in Supplementary Table 6. Raw dot plots for Rad21-HT are provided in Supplementary Fig. 15 and for HT-Rad21 in Supplemenarty Fig. 9. Source data are provided as a Source Data file.

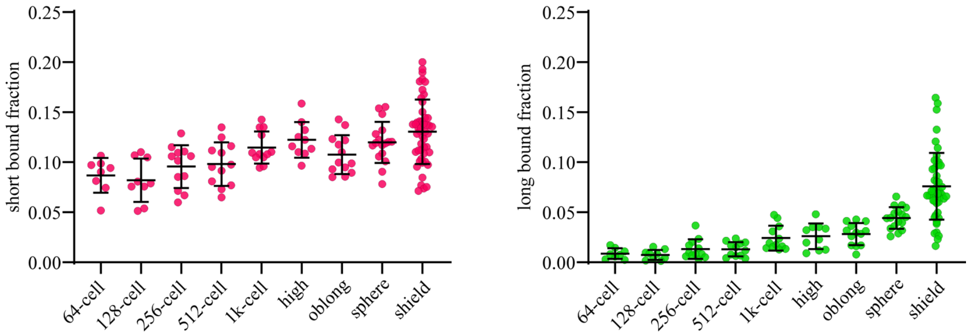

Supplementary Figure 15. Raw data plots for Rad21-HT. Raw data are shown for the fractions of short (left) and long (right) binding events of Rad21-HT recorded with interlaced time-lapse microscopy (ITM) illumination (compare Supplementary Fig. 14). Data represent mean ± s.d. Source data are provided as a Source Data file for the original Supplementary Fig. 14.

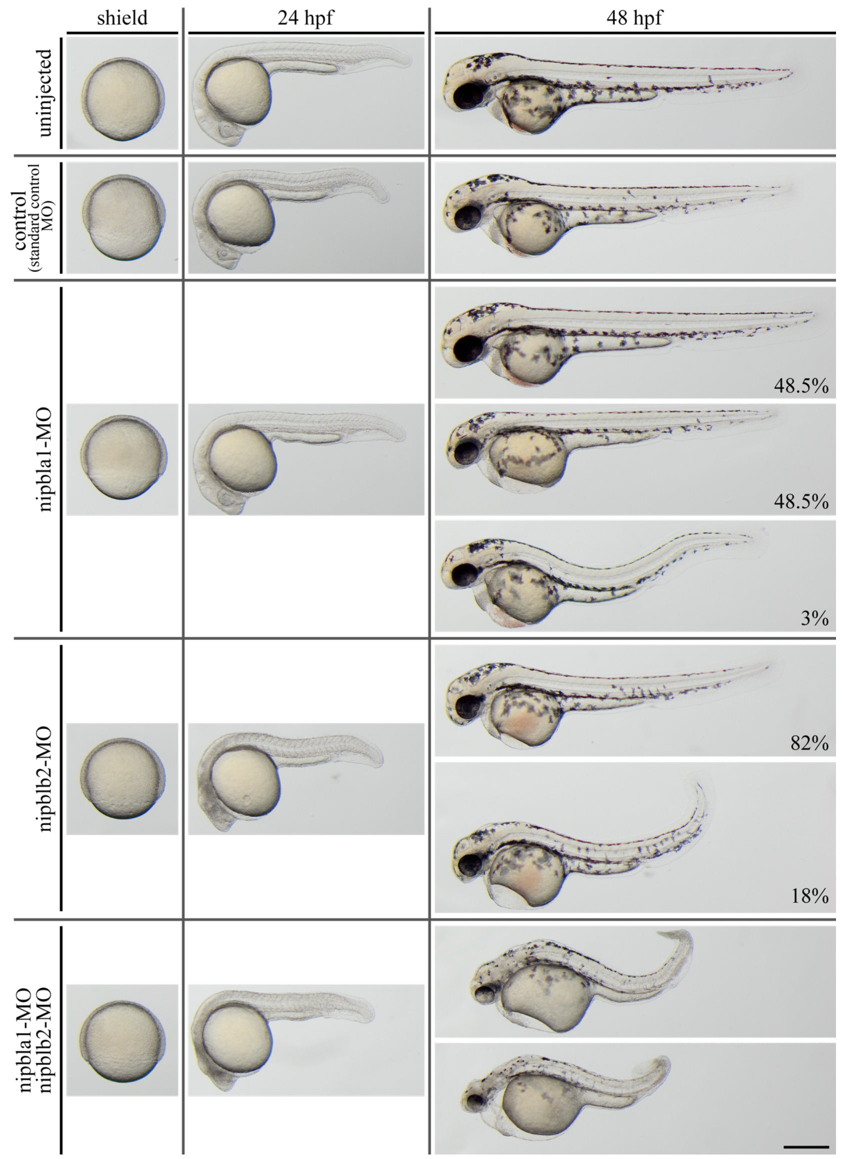

Supplementary Figure 16. Development of nipbl-morpholino (MO) injected zebrafish embryos. Lateral views of whole embryos either uninjected or injected with standard control MO (Gene Tools LLC , USA), nipbla1-MO, nipblb2-MO, nipbla1- and nipblb2-MO (see Methods). At 24 hours post-fertilization (hpf), nipblb2-MO and coinjected nipbla1/nipblb2-MO zebrafish exhibit shortened tails. By 48 hpf, each nipbl-MO and the coinjection show pericardial edema and tail defects. Coinjection additionally causes developmental defects and non-specific proliferation defects at 48 hpf. Images of coinjected fish at 48 hpf exemplify a range of phenotypes, including tail and proliferation defects up to necrosis. Stages are provided according to Kimmel et al., 1995. Scale bar is 500 µm and applies to all images.

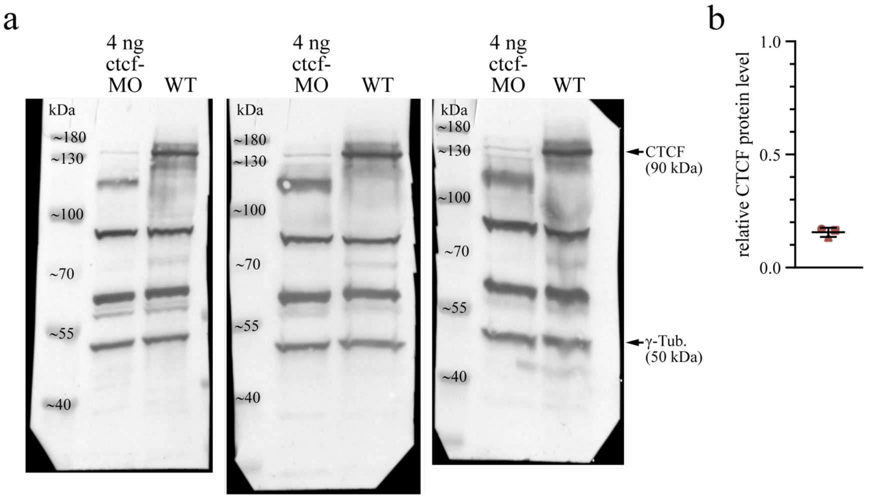

Supplementary Figure 17. Quantification of CTCF expression level after ctcf-morpholino (MO) injection. **a)** Western blots with anti-CTCF and anti-γ-Tubulin (γ-Tub.) antibodies of shield-stage zebrafish embryo lysate after injection of ctcf-MO. **b)** Quantification of CTCF protein level from ctcf-MO injected embryos compared to WT and loading control shown in a). Lines represent mean value ± s.d. Source data are provided as a Source Data file for Supplementary Fig.17b.

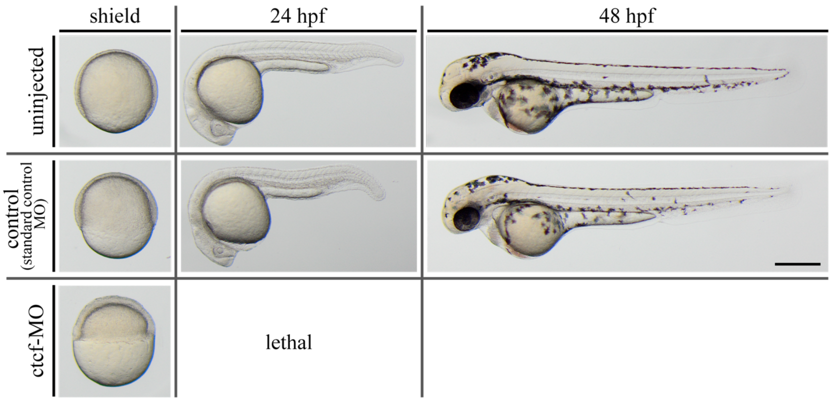

Supplementary Figure 18. Development of ctcf-morpholino (MO) injected zebrafish embryos. Lateral views of whole embryos either uninjected or injected with standard control MO (Gene Tools LLC, USA) or ctcf-MO (see Methods). Embryos injected with ctcf-MO exhibited developmental delays, remaining at around 50% epiboly, compared to the time point of shield stage in both uninjected and standard control-MO injected embryos. Injection of ctcf-MO was lethal beyond 24 hours post-fertilization (hpf). Stages are provided according to Kimmel et al., 1995. Scale bar is 500 µm and applies to all images.

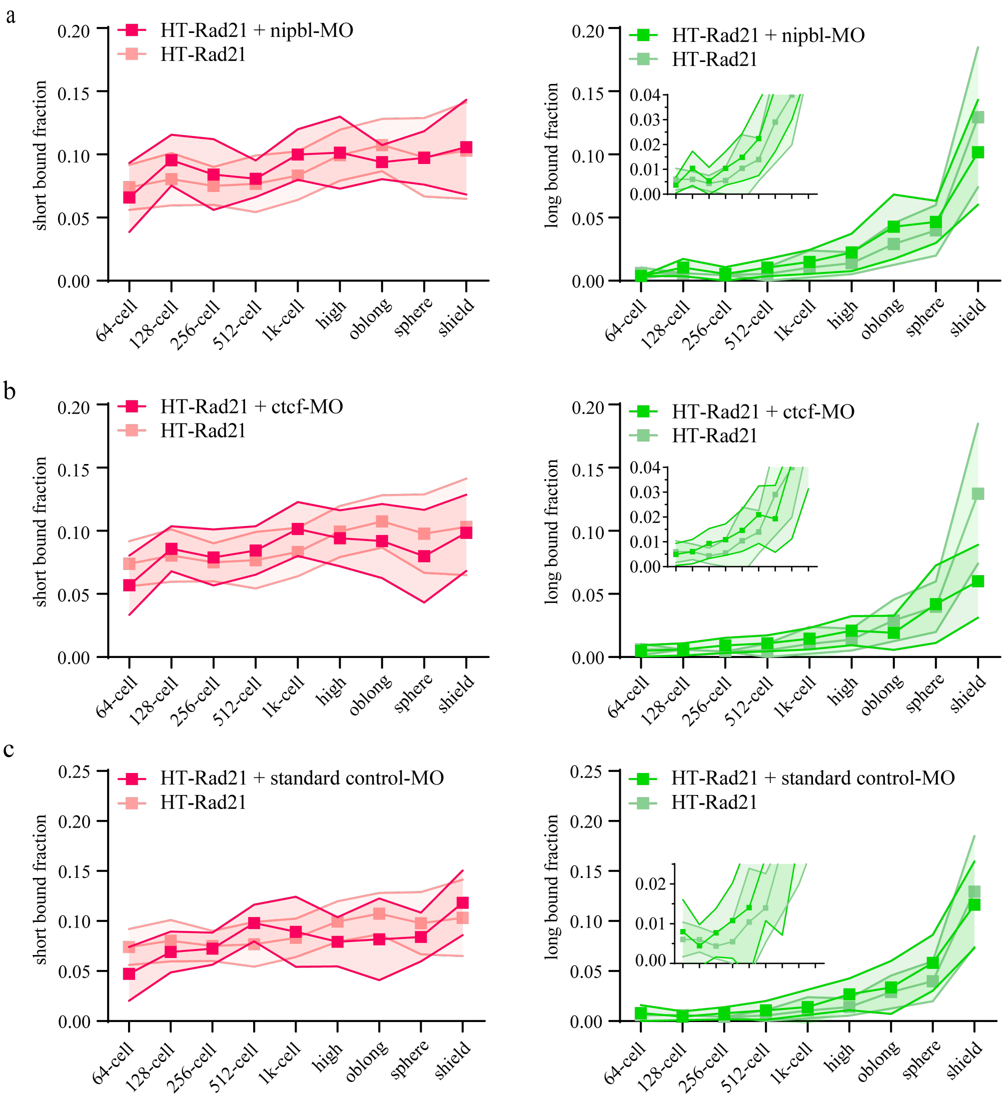

Supplementary Figure 19. Short and long-bound fractions of HT-Rad21 in presence of nipbl-morpholino (MO), ctcf-MO, or standard control MO. Fractions of short and long binding events from ITM measurements of HT-Rad21 in **a)** embryos injected with nipbl-MO (HT-Rad21 + nipbl-MO) or uninjected embryos (HT-Rad21), **b)** embryos injected with ctcf-MO (HT-Rad21 + ctcf-MO) or uninjected embryos (HT-Rad21) and **c)** embryos injected with standard control-MO (HT-Rad21 + standard control-MO) or uninjected embryos (HT-Rad21). Data represent mean ± s.d. of movie-wise determined fractions. Insets show zooms into the respective graphs. Lines serve as guides to the eye. Statistics and p-values fors MOs are provided in Supplementary Tables 21-22 and for HT-Rad21 in Supplementary Table 6. Raw dot plots for MOs are provided in Supplementary Fig. 20 and for HT-Rad21 in Supplementary Fig. 9. Source data are provided as a Source Data file.

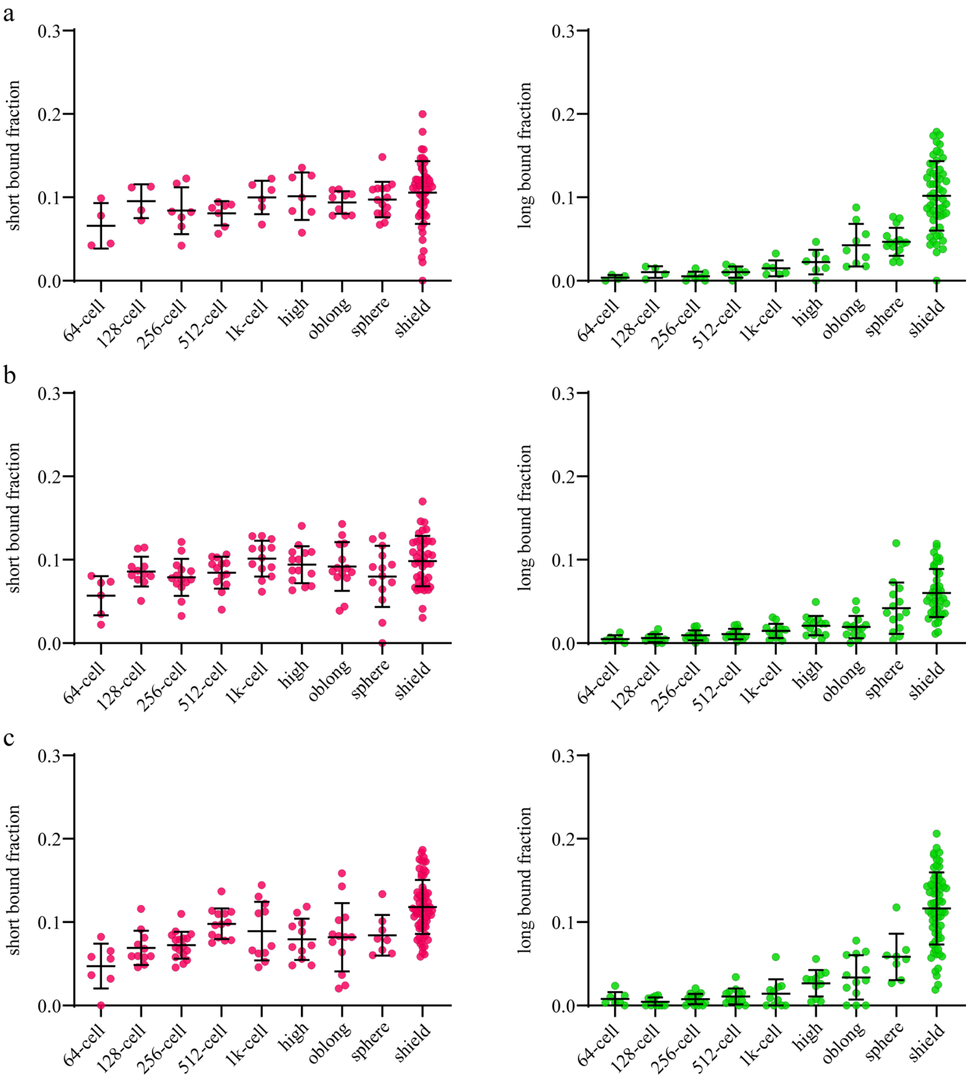

Supplementary Figure 20. Raw data plots for HT-Rad21 with coinjection of nipbl-morpholino (MO), ctcf-MO or standard control MO. Raw data are shown for the fractions of short (left) and long (right) binding events of **a)** HT-Rad21 + ctcf-MO, **b)** HT-Rad21 + nipbl-MO or **c)** HT-Rad21 with standard control MO injection recorded with interlaced time-lapse microscopy (ITM) (compare Supplementary Fig. 19). Data represent mean ± s.d. Source data are provided as a Source Data file for the original Supplementary Fig. 19.

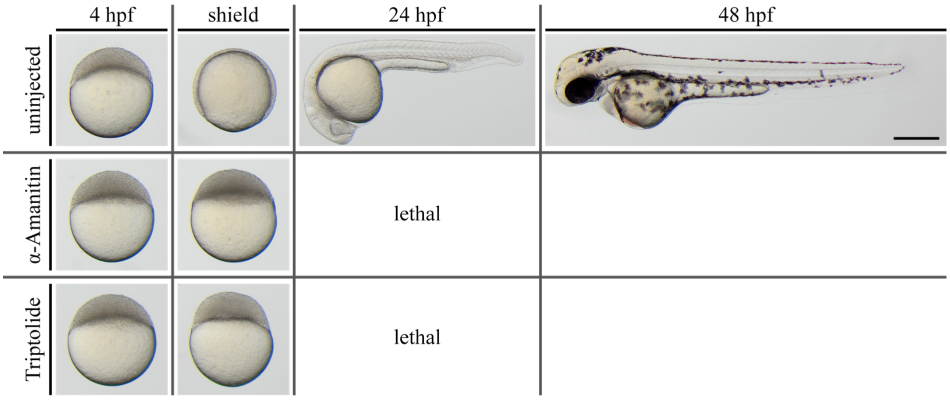

Supplementary Figure 21. Development of α-Amanitin and Triptolide treated zebrafish embryos. Lateral views of whole embryos either uninjected (WT) or treated with α-Amanitin or Triptolide (see Methods). Embryos injected with α-Amanitin or Triptolide exhibited developmental delays, remaining at around sphere stage, compared to the time point of shield stage in uninjected embryos. Both injections were lethal at 24 hours post-fertilization (hpf). Stages are provided according to Kimmel et al., 1995. Scale bar is 500 µm and applies to all images.

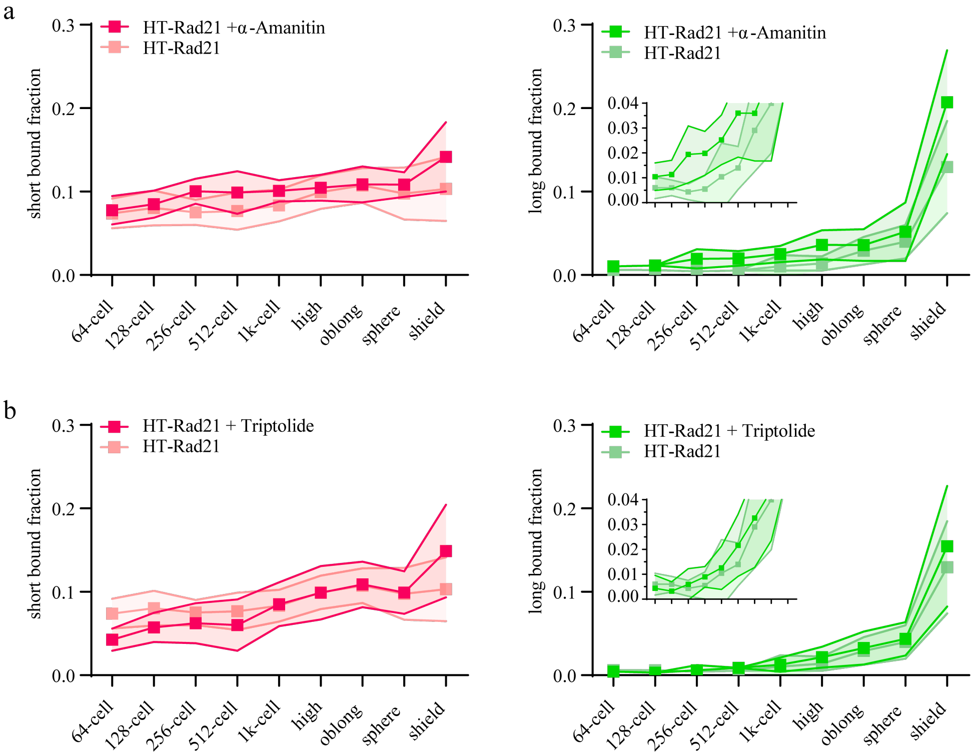

Supplementary Figure 22. Short and long-bound fractions of HT-Rad21 in presence of α-Amanitin or Triptolide. Fractions of short and long binding events from ITM measurements of HT-Rad21 in **a)** embryos treated with α-Amanitin (HT-Rad21 + α-Amanitin) or untreated embryos (HT-Rad21) and **b)** embryos treated with Triptolide (HT-Rad21 + Triptolide) or untreated embros (HT-Rad21). Data represent mean ± s.d. of movie-wise determined fractions. Insets show zooms into the respective graphs. Lines serve as guides to the eye. Statistics and p-values for both treatments are provided in Supplementary Tables 23-24 and for HT-Rad21 in Supplementary Table 6. Raw dot plots for both treatments are provided in Supplementary Fig. 23 and for HT-Rad21 in Supplementary Fig. 9. Source data are provided as a Source Data file.

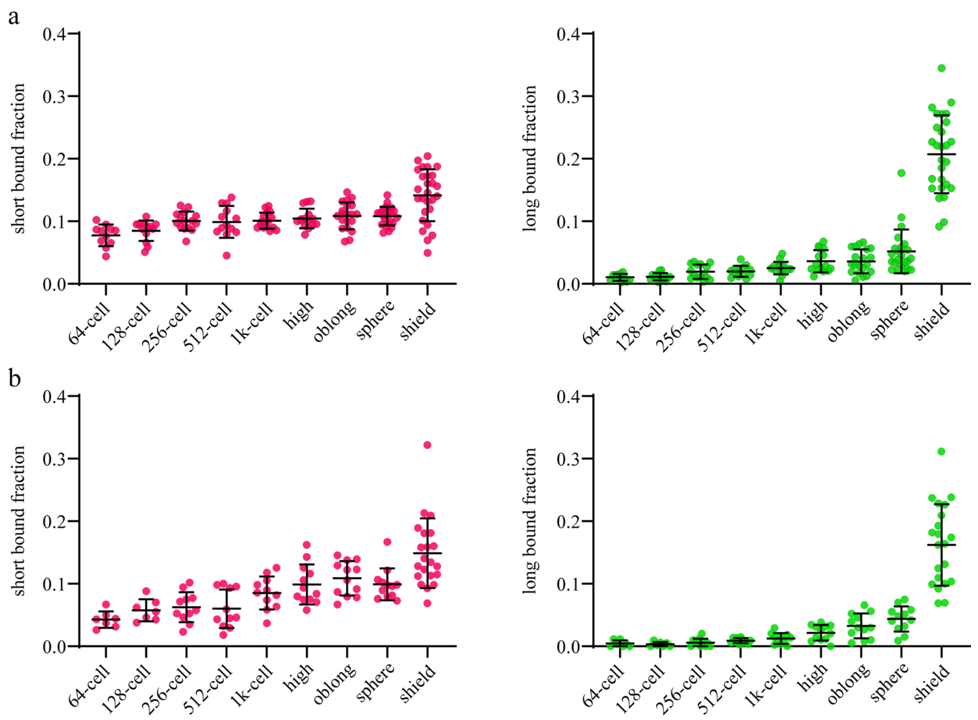

Supplementary Figure 23. Single dot plots for HT-Rad21 and RNA polymerase inhibitors α-Amanitin and Triptolide. a) Raw data are shown for the fractions of short (left) and long (right) binding events of **a)** HT-Rad21 + α-Amanitin and **b)** HT-Rad21 + Triptolide recorded with interlaced time-lapse microscopy (ITM) (compare Supplementary Fig. 22). Data represent mean ± s.d. Source data are provided as a Source Data file for the original Supplementary Fig. 22.

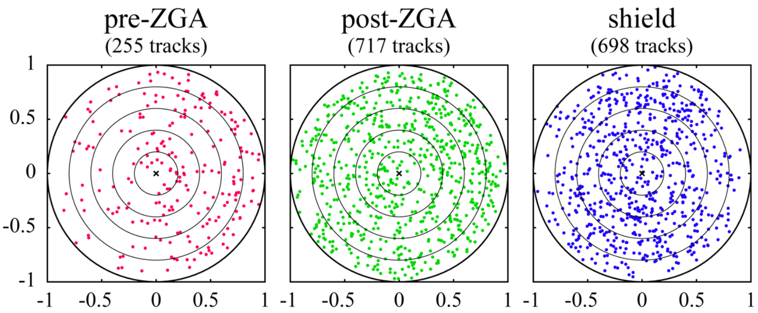

Supplementary Figure 24. Unit circles for Center-Border Distance (CBD) analysis of HT-Rad21 initial positions. Initial positions of tracks classified as long-bound in ITM measurements (Fig 1f) and TACO measurements (Fig 4a) from pooled stages are shown on a unit circle. pre-ZGA: 64-, 128-, 256, 512-cell stages pooled; post-ZGA: high, oblong, sphere stages pooled; shield stage.

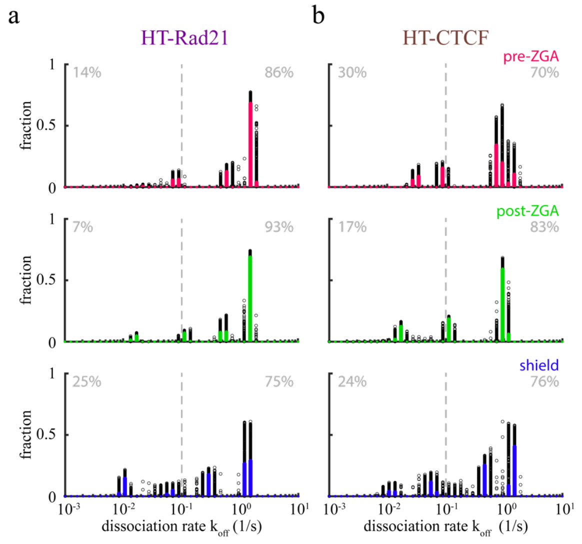

Supplementary Figure 25. Event spectra of dissociation rates. GRID event spectra of dissociation rates of **a)** HT-Rad21 and **b)** HT-CTCF using all data (solid line, colored according to stages) and 500 resampling runs with randomly selected 80% of data (black spots) as an error estimation of the spectra. Grey insets: Percentages of dissociation rates larger or smaller than 0.1 s^{-1} (dashed line). State spectra and residence times are provided in Fig. 3f and g. Statistics are provided in Supplementary Table 14. Source data are provided as a Source Data file.

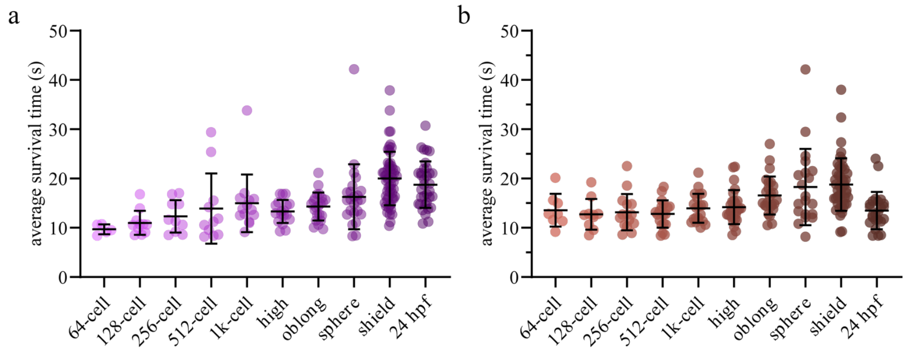

Supplementary Figure 26. Relative increase of survival times. Average survival times of binding events in interlaced time-lapse microscopy (ITM) that were classified as long-bound (>8.2 s) for **a**) HT-Rad21 or **b)** HT-CTCF molecules. All other tracks (<8.2 s) were not considered. Data represent mean ± s.d. of movie-wise averages. Source data are provided as a Source Data file.

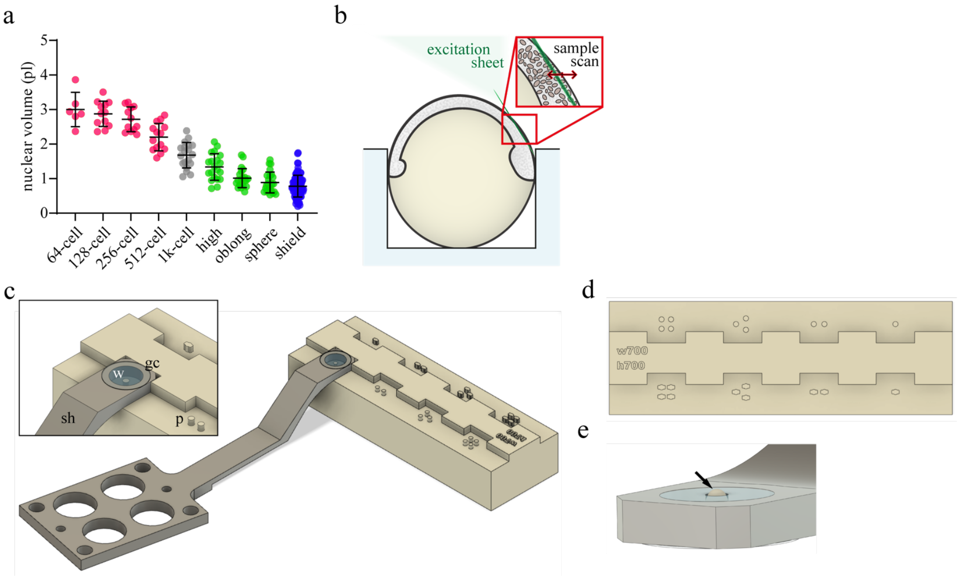

Supplementary Figure 27. Zebrafish nuclear volume and mounting of embryos on a lattice light-sheet microscope (LLSM). a) Nuclear volume at multiple stages of development (see Methods). Data represent mean ± s.d. **b)** Scheme of a shield stage zebrafish embryo placed in an agarose well to scan through nuclei with a LLSM. **c**) LLSM sample holder (grey) with a 5 mm coverslip glued on top and filled with agarose. A 3D-printed stamp (brown) with pins is used to form wells in the agarose. Inset: sample holder (sh), agarose well (w), glass coverslip (gc), pins (p). **d)** Top-down view of the 3D printed stamp with multiple pin combinations for early stages up to shield stage (top row) and later stages post 24 hpf (bottom row) **e)** Zoom-in on the LLSM sample holder filled with agarose, showing the animal cap of an embryo mounted in the center (arrow). The animal cap is above the agarose, allowing undisturbed excitation of fluorophores and emission of fluorescent light. Source data are provided as a Source Data file for Supplementary Fig. 27a.

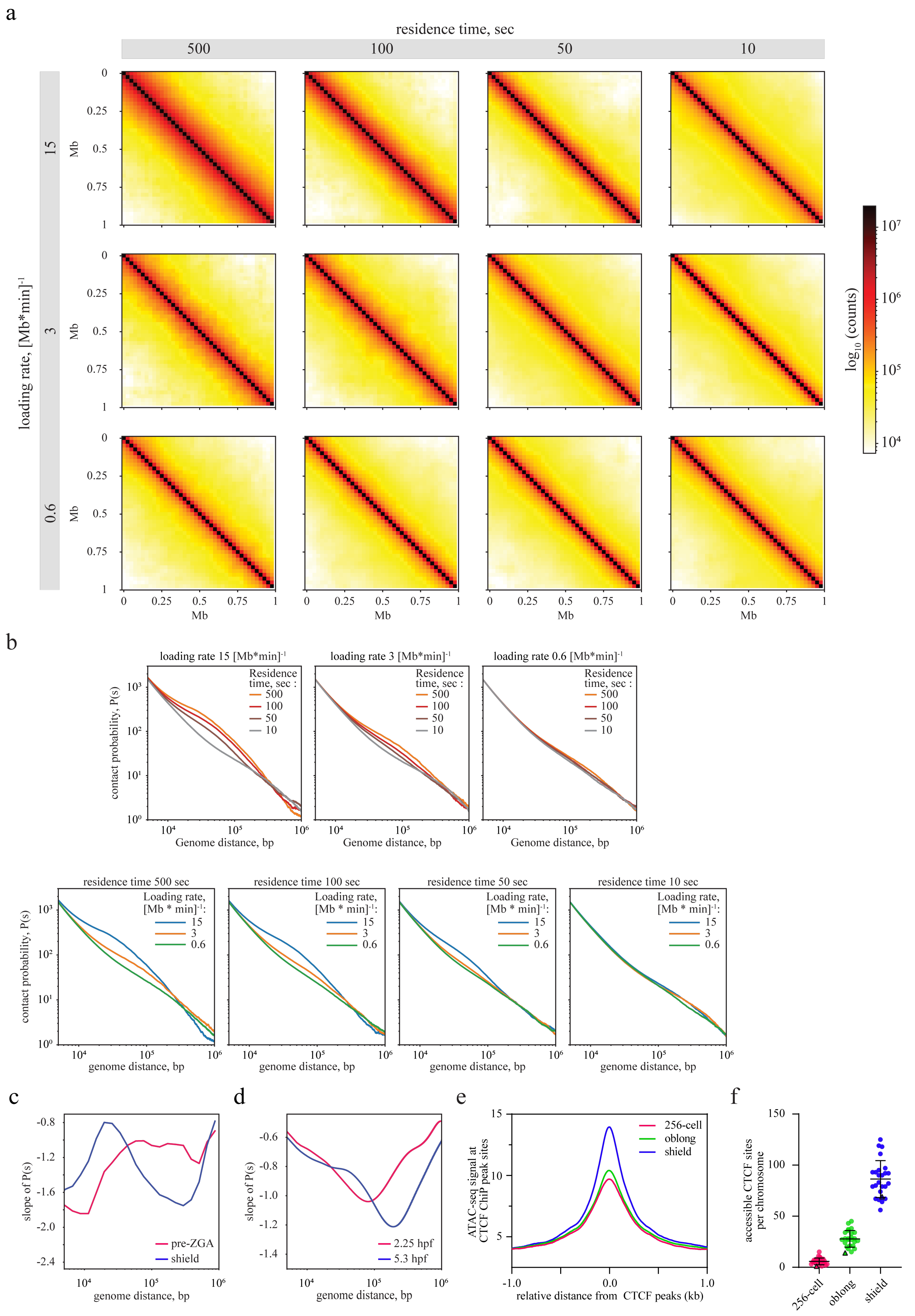

a
residence time, sec
500
100
50
10
loading rate, [Mb*min]-1
15
3
0.6
Mb
log10 (counts)
b
loading rate 15 [Mb*min]-1
loading rate 3 [Mb*min]-1
loading rate 0.6 [Mb*min]-1
Residence time, sec :
contact probability, P(s)
Genome distance, bp
residence time 500 sec
residence time 100 sec
residence time 50 sec
residence time 10 sec
Loading rate, [Mb * min]-1:
genome distance, bp
c
slope of P(s)
pre-ZGA
shield
d
2.25 hpf
5.3 hpf
e
ATAC-seq signal at CTCF ChiP peak sites
256-cell
oblong
shield
relative distance from CTCF peaks (kb)
f
accessible CTCF sites per chromosome
256-cell
oblong
shield

Supplementary Figure 28. Sweeping of parameter space, CTCF sites and Chromatin accessibility.
a) Contact maps of the polymer-chain model at 1 kb mapping. The parameter space of extruder loading rates and residence times covers 1-2 orders of magnitude. **b)** The dependence of contact probability P(s) on genomic distance for contact maps shown in panel a). **c)** Slopes of P(s) for pre-ZGA and shield stages from Fig. 4a. **d)** Slopes of P(s) for 2.25 hpf and 5.3 hpf from Fig. 4b. **e)** ATAC-seq profile for three developmental stages at CTCF sites identified by ChIP-seq based on published data[1,2] **f)** Distributions of accessible CTCF sites per chromosome identified in open chromatin regions by ATAC-seq for three developmental stages. Chr18 is highlighted with a triangle. Source data are provided as a Source Data file for Supplementary Fig. 28e and f.

Supplementary Tables

	HT-Rad21					HT-CTCF				
	Days	Embryo	Movie	Tracks	Jumps	Days	Embryo	Movie	Tracks	Jumps
64-cell	4	7	10	1474	8432	2	4	9	1654	8992
128-cell	4	9	13	1828	9744	2	6	10	1455	7675
256-cell	4	8	13	1939	12222	2	7	14	1859	10060
512-cell	4	12	16	1697	11517	2	7	13	1616	10406
1k-cell	4	11	21	1888	12932	2	8	15	1900	12585
high	4	9	14	1376	10613	2	8	16	1773	11856
oblong	4	6	11	1123	9049	2	7	15	1507	10915
sphere	4	9	21	1391	12035	2	7	20	1870	15339
shield	3	12	29	922	9637	2	9	35	2422	17525

Supplementary Table 1. Statistics of 11.7 ms continuous movies recorded for HT-Rad21 and HT-CTCF. Related to Fig. 1d,e.

	long-bound fraction	short-bound fraction
64-cell	0.0030	0.1079
128-cell	0.0002	0.0409
256-cell	0.0032	0.0409
512-cell	0.0388	0.7267
1k-cell	0.0051	0.2864
high	<0.0001	0.1968
oblong	<0.0001	0.1762
sphere	<0.0001	0.4819
shield	<0.0001	0.1645
24 hpf	<0,0001	0.2345

Supplementary Table 2. P-values from two-sided Multiple Mann-Whitney test with a false discovery rate set to 1% on bound fractions of HT-Rad21 vs. HT-Rad21-3x, based on interlaced time-lapse microscopy (ITM) data shown in Fig. 1h.

	long-bound fraction	short-bound fraction
64-cell	0.0009	0.7802
128-cell	0.0002	0.6522
256-cell	<0.0001	0.0105
512-cell	<0.0001	0.0361
1k-cell	<0.0001	0.0220
high	<0.0001	0.2041
oblong	<0.0001	0.0093
sphere	<0.0001	0.0133
shield	<0.0001	0.0008
24 hpf	<0.0001	0.6419

Supplementary Table 3. P-values from two-sided Multiple Mann-Whitney test with a false discovery rate set to 1% on bound fractions of HT-CTCF vs. HT-CTCF-ΔZF4-7, based on interlaced time-lapse microscopy (ITM) data shown in Fig. 1g.

	long-bound fractions								
	128-cell	256-cell	512-cell	1k-cell	high	oblong	sphere	shield	24 hpf
64-cell	>0.9999	>0.9999	>0.9999	>0.9999	>0.9999	>0.9999	0.8898	0.0001	<0.0001
128-cell		>0.9999	>0.9999	>0.9999	>0.9999	0.8844	0.1341	<0.0001	<0.0001
256-cell			>0.9999	>0.9999	>0.9999	0.2909	0.0343	<0.0001	<0.0001
512-cell				>0.9999	>0.9999	0.3452	0.0373	<0.0001	<0.0001
1k-cell					>0.9999	0.9325	0.1024	<0.0001	<0.0001
high						>0.9999	>0.9999	<0.0001	<0.0001
oblong							>0.9999	0.0002	<0.0001
sphere								0.0044	0.0003
shield									>0.9999

	short-bound fractions								
	128-cell	256-cell	512-cell	1k-cell	high	oblong	sphere	shield	24 hpf
64-cell	>0.9999	>0.9999	>0.9999	>0.9999	>0.9999	0.2965	>0.9999	0.8475	0.0590
128-cell		>0.9999	>0.9999	>0.9999	>0.9999	0.4666	>0.9999	>0.9999	0.0517
256-cell			>0.9999	>0.9999	0.4719	0.0236	>0.9999	0.0732	0.0011
512-cell				>0.9999	>0.9999	0.0592	>0.9999	0.1878	0.0029
1k-cell					>0.9999	0.3497	>0.9999	>0.9999	0.0202
high						>0.9999	>0.9999	>0.9999	>0.9999
oblong							>0.9999	>0.9999	>0.9999
sphere								>0.9999	0.7912
shield									>0.9999

Supplementary Table 4. P-values from two-sided Kruskal-Wallis test followed by Dunn's multiple comparison test on bound fractions of HT-Rad21, based on interlaced time-lapse microscopy (ITM) data shown in Fig. 1h.

	long-bound fractions								
	128-cell	256-cell	512-cell	1k-cell	high	oblong	sphere	shield	24 hpf
64-cell	>0.9999	>0.9999	>0.9999	>0.9999	0.1876	0.0005	0.0019	0.0007	0.0008
128-cell		>0.9999	>0.9999	0.8534	0.0371	<0.0001	0.0002	<0.0001	<0.0001
256-cell			>0.9999	>0.9999	0.2592	0.0002	0.0012	0.0002	0.0003
512-cell				>0.9999	>0.9999	0.0121	0.0490	0.0133	0.0195
1k-cell					>0.9999	0.0804	0.2552	0.1060	0.1213
high						>0.9999	>0.9999	>0.9999	>0.9999
oblong							>0.9999	>0.9999	>0.9999
sphere								>0.9999	>0.9999
shield									>0.9999

	short-bound fractions								
	128-cell	256-cell	512-cell	1k-cell	high	oblong	sphere	shield	24 hpf
64-cell	>0.9999	>0.9999	0.5778	0.1195	0.1444	0.3779	0.0147	0.0005	0.0009
128-cell		>0.9999	>0.9999	0.9423	>0.9999	>0.9999	0.1467	0.0069	0.0117
256-cell			>0.9999	>0.9999	>0.9999	>0.9999	>0.9999	0.4751	0.5842
512-cell				>0.9999	>0.9999	>0.9999	>0.9999	>0.9999	>0.9999
1k-cell					>0.9999	>0.9999	>0.9999	>0.9999	>0.9999
high						>0.9999	>0.9999	>0.9999	>0.9999
oblong							>0.9999	0.8510	>0.9999
sphere								>0.9999	>0.9999
shield									>0.9999

Supplementary Table 5. P-values from two-sided Kruskal-Wallis test followed by Dunn's multiple comparison test on bound fractions of HT-CTCF, based on interlaced time-lapse microscopy (ITM) data shown in Fig. 1g.

	HT-Rad21				HT-Rad21-3x mutant			
	Days	Embryos	Movies	Mean number of all events per movie	Days	Embryos	Movies	Mean number of all events per movie
64-cell	4	6	6	752	2	6	8	427
128-cell	4	8	12	676	2	6	9	541
256-cell	4	8	12	573	2	5	9	421
512-cell	4	9	14	506	2	6	11	366
1k-cell	4	10	19	429	2	6	10	372
high	4	11	20	399	2	6	11	293
oblong	4	11	22	331	2	6	10	230
sphere	4	9	24	245	2	6	14	149
shield	2	9	56	68	2	8	38	40
24 hpf	2	11	34	45	2	6	14	105

Supplementary Table 6. Statistics of interlaced time-lapse microscopy (ITM) movies recorded for HT-Rad21 and HT-Rad21-3x. Related to ITM data in Fig. 1h.

	HT-CTCF				HT-CTCF-ΔZF4-7 mutant			
	Days	Embryos	Movies	Mean number of all events per movie	Days	Embryos	Movies	Mean number of all events per movie
64-cell	3	5	9	284	2	6	10	718
128-cell	4	6	11	264	2	7	11	769
256-cell	4	7	16	241	2	7	11	796
512-cell	5	10	20	245	2	7	14	813
1k-cell	5	8	21	256	2	6	11	673
high	5	10	26	211	2	6	11	666
oblong	5	9	25	175	2	6	13	555
sphere	3	6	20	133	2	6	12	452
shield	2	11	46	195	2	9	50	324
24 hpf	2	7	24	92	2	7	23	184

Supplementary Table 7. Statistics of interlaced time-lapse microscopy (ITM) movies recorded for HT-CTCF and HT-CTCF-ΔZF4-7. Related to ITM data in Fig. 1g.

HT-Rad21 +	**short-bound**	**long-bound**
nipbl-MO	0.5724	0.0108
ctcf-MO	0.4907	<0.0001
α-Amanitin	<0.0001	<0.0001
Triptolide	0.0004	0.0538

Supplementary Table 8. P-values from two-sided Multiple Mann-Whitney test with a false discovery rate set to 1% on bound fractions of HT-Rad21 wild-type vs. morpholino addition (MO) or RNA-Polymerase inhibitors, based on interlaced time-lapse microscopy (ITM) data shown in Fig. 1i. Full statistical tests on all stages (Supplementary Fig. 19 and 22) are provided in Supplementary Table 21 and 23.

(shield stage) HT-Rad21+	Days	Embryos	Movies	Mean number of all events per movie
nipbl-MO	2	9	54	94
ctcf-MO	2	5	41	122
α-Amanitin	2	4	27	126
Triptolide	2	9	21	96

Supplementary Table 9. Statistics of bound fractions of HT-Rad21 wild-type vs. morpholino addition (MO) or RNA-Polymerase Inhibitors for interlaced time-lapse microscopy (ITM) measurements. Related to Fig. 1i.

		**short-bound**		
		post-ZGA	shield	shield + nibpl-MO
**short-bound**	pre-ZGA	>0.9999	<0.0001	<0.0001
	post-ZGA		<0.0001	0.0002
	shield			>0.9999

		**long-bound**		
		post-ZGA	shield	shield + nibpl-MO
**long-bound**	pre-ZGA	0.0003	<0.0001	<0.0001
	post-ZGA		0.0023	>0.9999
	shield			0.0011

**long-bound vs. short-bound**	
pre-ZGA	<0.0001*
post-ZGA	<0.0001*
shield	<0.0001*
shield + nibpl-MO	<0.0001*

Supplementary Table 10. P-values from two-sided Kruskal-Wallis test followed by Dunn's multiple comparison test and two-sided Multiple Mann-Whitney test (*) with a false discovery rate set to 1% on mean jump distances of HT-Rad21 and morpholino addition (MO), based on time-lapse alternated with continuous intervals (TACO) data shown in Fig. 2b.

		Days	Embryos	Movies	Tracks
short-bound	pre-ZGA	3	8	36	200
	post-ZGA	3	7	36	221
	shield	4	15	43	135
	shield +nibpl-MO	3	12	35	166
long-bound	pre-ZGA	4	11	33	80
	post-ZGA	4	11	41	173
	shield	4	15	42	248
	shield +nibpl-MO	3	13	41	412

Supplementary Table 11. Statistics of time-lapse alternated with continuous intervals (TACO) movies recorded for HT-Rad21. Related to Fig. 2b.

		Cluster			
		#1 (longest)	#2	#3	#4 (shortest)
pre-ZGA	residence time of cluster ± s.d. of resampling (s)	52.08 (± 5.7)	12.74 (± 0.62)	1.66 (± 0.14)	0.65 (± 0.03)
	fraction of cluster ± s.d. of resampling	25.3 (± 3.3)	52.2 (± 3)	7.39 (± 1.2)	15.1 (± 0.89)
post-ZGA	residence time of cluster ± s.d. of resampling (s)	62.89 (± 1.9)	8.62 (± 0.59)	1.88 (± 0.17)	0.67 (± 0.01)
	fraction of cluster ± s.d. of resampling	74.5 (± 1.1)	12.2 (± 0.75)	5.33 (± 0.63)	8.02 (± 0.45)
shield	residence time of cluster ± s.d. of resampling (s)	99.01 (± 5.69)	15.92 (± 3.55)	3.53 (± 0.42)	0.75 (± 0.06)
	fraction of cluster ± s.d. of resampling	88.6 (± 1.9)	6.05 (± 1.7)	3.27 (± 0.58)	2.12 (± 0.17)

Supplementary Table 12. Residence times of HT-Rad21. Residence times obtained from dissociation rate clusters of GRID state spectra shown in Fig. 3f.

		Cluster		
		#1 (longest)	#2	#3 (shortest)
pre-ZGA	residence time of cluster ± s.d. of resampling (s)	32.57 (± 1.8)	11.52 (± 0.94)	1.13 (± 0.04)
	fraction of cluster ± s.d. of resampling	63.4 (± 4.1)	26 (± 4)	10.6 (± 0.73)
post-ZGA	residence time of cluster ± s.d. of resampling (s)	61.35 (± 2.63)	9.01 (± 0.48)	1.04 (± 0.04)
	fraction of cluster ± s.d. of resampling	79.1 (± 1.2)	14.9 (± 0.94)	5.94 (± 0.39)
shield	residence time of cluster ± s.d. of resampling (s)	88.5 (± 10.96)	17.33 (± 1.83)	1.19 (± 0.07)
	fraction of cluster ± s.d. of resampling	65.3 (± 4.2)	26.6 (± 4)	8.09 (± 1.1)

Supplementary Table 13. Residence times of HT-CTCF. Residence times obtained from dissociation rate clusters of GRID state spectra shown in Fig. 3g.

		Days	Embryos	Movies	Tracks	Tracks per movie
HT-Rad21 502ms	preZGA	3	10	62	1582	25.5
	postZGA	3	9	59	2580	43.7
	shield	3	10	68	1561	23.0
HT-Rad21 4.5s	preZGA	4	9	16	769	48.1
	postZGA	4	12	23	1272	55.3
	shield	4	10	22	517	23.5
HT-CTCF 502ms	preZGA	4	11	33	2334	70.7
	postZGA	4	11	24	1486	61.9
	shield	4	15	18	1278	71.0
HT-CTCF 4.5s	preZGA	4	10	16	1734	108.4
	postZGA	4	10	22	1576	71.6
	shield	4	8	11	774	70.4

Supplementary Table 14. Statistics of time-lapse microscopy movies recorded for HT-Rad21 and HT-CTCF. Related to Fig. 3d-g.

	$k_{off,u}$	$A^e{}_s$	$A^s{}_u$	f_b
pre-ZGA	0.835	0.30	0.12	0.20
post-ZGA	0.386	0.17	0.19	0.21
shield	0.792	0.24	0.09	0.23

Supplementary Table 15. Measured parameters used to calculate the search time τ_{search} for a single HT-CTCF molecule. Related to Fig. 3i and Methods.

	$k_{off,s}$	$A^s{}_s$	f_b
pre-ZGA	0.0591	0.77	0.05
post-ZGA	0.0165	0.75	0.08
shield	0.0132	0.95	0.31

Supplementary Table 16. Measured parameters used to calculate the search time τ_{search} for a single HT-Rad21 molecule. Related to Fig. 3i and Methods.

	**HT control vs HT-Rad21**		**HT control vs HT-CTCF**	
	short-bound	long-bound	short-bound	long-bound
64-cell	0.0002	0.0014	0.0002	0.0001
128-cell	<0.0001	<0.0001	<0.0001	<0.0001
256-cell	<0.0001	<0.0001	<0.0001	<0.0001
512-cell	<0.0001	<0.0001	<0.0001	<0.0001
1k-cell	<0.0001	0.0001	<0.0001	<0.0001
high	<0.0001	<0.0001	<0.0001	<0.0001
oblong	<0.0001	<0.0001	<0.0001	<0.0001
sphere	<0.0001	<0.0001	<0.0001	<0.0001
shield	<0.0001	<0.0001	<0.0001	<0.0001

Supplementary Table 17. P-values from two-sided Multiple Mann-Whitney test with a false discovery rate set to 1% comparing bound fractions of HT control vs HT-Rad21 or HT-CTCF, based on interlaced time-lapse microscopy (ITM) data shown in Supplementary Fig. 10.

	**HT only**			
	Days	Embryos	Movies	Mean number of all events per movie
64-cell	3	5	10	216
128-cell	3	7	15	193
256-cell	3	7	16	179
512-cell	3	7	19	194
1k-cell	3	6	16	165
high	3	7	16	164
oblong	3	6	13	148
sphere	3	4	11	143
shield	3	10	34	89

Supplementary Table 18. Statistics of interlaced time-lapse microscopy (ITM) movies recorded for HT-control. Related to Supplementary Fig. 10.

	Rad21-HT vs HT-Rad21	
	short-bound	long-bound
64-cell	0.2284	0.3450
128-cell	0.8078	0.4639
256-cell	0.0100	0.0023
512-cell	0.0270	0.0054
1k-cell	<0.0001	0.0007
high	0.0146	0.0143
oblong	0.9329	0.7748
sphere	0.0044	0.4550
shield	0.0002	<0.0001

Supplementary Table 19. P-values from two-sided Multiple Mann-Whitney test with a false discovery rate set to 1% comparing bound fractions of Rad21-HT vs HT-Rad21, based on interlaced time-lapse microscopy (ITM) data shown in Supplementary Fig. 14.

	Rad21-HT			
	Days	Embryos	Movies	Mean number of all events per movie
64-cell	2	4	8	840
128-cell	2	6	9	751
256-cell	2	6	12	722
512-cell	2	6	12	677
1k-cell	2	6	12	559
high	2	5	10	505
oblong	2	6	13	421
sphere	2	6	17	342
shield	2	8	49	162

Supplementary Table 20. Statistics of interlaced time-lapse microscopy (ITM) movies recorded for Rad21-HT. Related to Supplementary Fig. 14.

	HT-Rad21 + ctcf-MO vs HT-Rad21		HT-Rad21 + nipbl-MO vs HT-Rad21		HT-Rad21 vs HT-Rad21 + Standard control MO	
	short-bound	long-bound	short-bound	long-bound	short-bound	long-bound
64-cell	0.3939	0.3290	0.6095	0.5143	0.1375	0.8718
128-cell	0.8328	0.9278	0.3791	0.2615	0.0908	0.5596
256-cell	0.5604	0.0107	0.4320	0.9500	0.7438	0.1472
512-cell	0.2020	0.0254	0.5846	0.1315	0.0291	0.1036
1k-cell	0.0369	0.0445	0.0690	0.1544	0.9662	0.6363
high	0.3587	0.0953	0.7253	0.1452	0.0255	0.0250
oblong	0.1089	0.0569	0.0636	0.2193	0.0135	0.6736
sphere	0.2475	0.8227	0.7482	0.4153	0.2731	0.0524
shield	0.4907	<0.0001	0.5724	0.0108	0.0410	0.3158

Supplementary Table 21. P-values from two-sided Multiple Mann-Whitney test with a false discovery rate set to 1% comparing bound fractions of HT-Rad21 + ctcf-MO vs HT-Rad21, HT-Rad21 + nipbl-MO vs HT-Rad21, and HT-Rad21 vs HT-Rad21 with standard control morpholino (MO), based on interlaced time-lapse microscopy (ITM) data shown in Supplementary Fig. 19.

HT-Rad21 +	ctcf-MO				nipbl-MO				HT-Rad21 + Standard control MO			
	Days	Embryos	Movies	Mean number of all events per movie	Days	Embryos	Movies	Mean number of all events per movie	Days	Embryos	Movies	Mean number of all events per movie
64-cell	2	4	6	312	2	3	4	546	3	5	7	202
128-cell	2	6	11	444	1	2	4	695	3	6	11	265
256-cell	2	7	14	384	2	4	7	476	3	7	17	318
512-cell	2	7	13	416	2	4	7	434	3	6	13	332
1k-cell	2	7	13	383	2	4	6	479	2	5	11	286
high	2	7	14	352	2	4	7	385	2	4	11	216
oblong	2	7	14	247	2	5	9	316	3	5	13	192
sphere	2	5	14	189	2	6	15	227	3	5	8	138
shield	2	5	41	122	2	9	54	94	5	20	66	144

Supplementary Table 22. Statistics of interlaced time-lapse microscopy (ITM) movies recorded for HT-Rad21 + ctcf-MO, HT-Rad21 + nipbl-MO, nd HT-Rad21 + standard control morpholino (MO). Related to Supplementary Fig. 19.

	HT-Rad21 + α-Amanitin vs HT-Rad21		HT-Rad21 + Triptolide vs HT-Rad21	
	short-bound	long-bound	short-bound	long-bound
64-cell	0.8075	0.1490	0.0047	0.3089
128-cell	0.4371	0.0160	0.0358	0.1234
256-cell	0.0003	<0.0001	0.1144	0.4361
512-cell	0.0255	<0.0001	0.2441	0.0578
1k-cell	0.0023	0.0003	0.8324	0.1874
high	0.5260	<0.0001	0.7737	0.0651
oblong	0.8323	0.1717	0.9011	0.6314
sphere	0.0570	0.5062	0.8817	0.5848
shield	<0.0001	<0.0001	0.0004	0.0538

Supplementary Table 23. P-values from two-sided Multiple Mann-Whitney test with a false discovery rate set to 1% comparing bound fractions of HT-Rad21 + α-Amanitin or HT-Rad21 + Triptolide vs HT-Rad21, based on interlaced time-lapse microscopy (ITM) data shown in Supplementary Fig. 22.

HT-Rad21 +	α-Amanitin				HT-Rad21 + Triptolide			
	Days	Embryos	Movies	Mean number of all events per movie	Days	Embryos	Movies	Mean number of all events per movie
64-cell	2	6	11	697	2	4	7	246
128-cell	2	6	13	780	2	4	7	293
256-cell	2	7	15	728	2	6	11	359
512-cell	2	6	13	625	2	6	11	314
1k-cell	2	7	16	621	2	6	11	356
high	2	7	15	439	2	6	12	277
oblong	2	7	20	399	2	6	12	279
sphere	2	7	23	308	2	5	12	245
shield	2	4	27	126	2	9	21	96

Supplementary Table 24. Statistics of interlaced time-lapse microscopy (ITM) movies recorded for HT-Rad + α-Amanitin or HT-Rad21 + Triptolide. Related to Supplementary Fig. 22.

CTCF fwd PacI	GAACTTTAATTAATATGGAAGGGGGACCGAC
CTCF rev AscI	GAACTGGCGCGCCTCACCGGTCCATCATGCTAAG
rad21a fwd PacI	GAACTTTAATTAAGATGTTTTACGCCCACTTCGTC
rad21a rev AscI	GAACTGGCGCGCCCTATACAATGTGGAAGCGTGGT
rad21a 3x fwd Q5SDM	GCAGCAGGCCATCGACCTGACGAAGACCGAGCCCTACAGTGAC
rad21a 3x rev Q5SDM	TTCTTCAGCACCCGGAAGCTGTAACGCTTGGCCGCAGCCTGTTT
CTCF ΔZF47 fwd Q5SDM:	AGAAAGTGCCGTTACTGTG
CTCF ΔZF47 rev Q5SDM:	CGGTTTCTCATGAGTGTG

Supplementary Table 25. Primers for cloning of HT constructs and Q5 Site-Directed Mutagenesis (Q5SDM).

	Frame cycle time (ms)	Excitation sequence
11.7 ms continuous	11.7	117 ms/10f 488, 1.17 s/100f 561
Interlaced time-lapse microscopy (ITM)	11.7	11x (2x (11.7 ms/1f 561, 117 ms/10f 488, 70.2 ms dark), 1.80 s dark, 117 ms/10f 488, 1.87 s dark)
0.5 s continuous time-lapse	501.7	501.7 ms/1f 488, 60.20 s/120f 561, 501.7 ms/1f 488
4.5 s time-lapse	501.7	501.7 ms/1f 561, 501.7 ms/1f 488, 3.51 s dark
Time-lapse alternated with continuous intervals (TACO) short	11.7	11x (11.7 ms/1f 561, 117 ms/10f 488, 70.2 ms dark, 117 ms/10f 561, 117 ms/10f 488, 3.76 s dark)
TACO long	11.7	3.6x (2x (11.7 ms /1f 561, 117 ms/10f 488, 4.06 s dark), 117 ms/10f 561, 1.92 s dark, 117 ms/10f 488, 2.04 s dark)

Supplementary Table 26. Illumination scheme names, frame cycle times (= exposure times + 1.7 ms readout time), and excitation sequences are given in time and frame counts (f), followed by the excitation laser wavelengths (488 nm, 561 nm).

	Construct	Threshold factor	Tracking radius (µm)	Min track length	Gap frames	Min. track length before gap frame
11.7 ms continuous	HT-Rad21, HT-CTCF	1	0.747	2	1	2
Interlaced time-lapse microscopy (ITM)	HT-Rad21, HT-Rad21-3x, HT-CTCF, HT-CTCF-ΔZF4-7	1	0.498	2	0	0
0.5 s continuous time-lapse	HT-Rad21, HT-CTCF	1	0.370	3	1	2
4.5 s time-lapse	HT-Rad21, HT-CTCF	1	0.907	3	1	2
Time-lapse alternated with continuous intervals (TACO) short	HT-Rad21	1	0.747	2	1	2
TACO long	HT-Rad21	1	0.747	2	1	2

Supplementary Table 27. Tracking parameters used in TrackIt[3] for our illumination schemes, all using the nearest neighbor algorithm.

>GCCWGCAGGGGGCGCTGSDG CTCF_zebrafish 3.643980 -616.688268 0 T:1993.0(42.57%),B:7136.0(20.02%),P:1e-267			
0.125	0.078	0.550	0.247
0.047	0.576	0.309	0.068
0.046	0.908	0.015	0.031
0.453	0.046	0.171	0.330
0.015	0.108	0.876	0.001
0.061	0.892	0.046	0.001
0.953	0.001	0.015	0.031
0.001	0.001	0.997	0.001
0.123	0.001	0.861	0.015
0.078	0.123	0.732	0.067
0.001	0.001	0.997	0.001
0.015	0.001	0.969	0.015
0.001	0.997	0.001	0.001
0.202	0.015	0.782	0.001
0.001	0.860	0.124	0.015
0.217	0.281	0.062	0.440
0.092	0.092	0.815	0.001
0.123	0.396	0.357	0.124
0.283	0.157	0.219	0.341
0.062	0.219	0.611	0.108

Supplementary Table 28. CTCF motif for CTCF orientation analysis. See Methods. Motif from a published dataset [2] with GEO accession number GSE133437.

Supplementary Movie Legends

File name: Supplementary Movie 1
Description: Example movie of HT-Rad21 mobility in 64-cell stage zebrafish embryo. Left: single molecule movie of HT-Rad21 molecules with continuous illumination at 11.7 ms frame cycle time (see Fig. 1c). Right: signal of the Lap2β nuclear membrane marker. Scale bar: 5 µm.

File name: Supplementary Movie 2
Description: Example movie of HT-Rad21 mobility in shield stage zebrafish embryo**.** Left: single molecule movie of HT-Rad21 molecules with continuous illumination at 11.7 ms frame cycle time (see Fig. 1c). Right: signal of the Lap2β nuclear membrane marker. Scale bar: 5 µm.

File name: Supplementary Movie 3
Description: Example movie of HT-Rad21 binding classes in 64-cell stage zebrafish embryos. Left: single molecule movie of HT-Rad21 molecules recorded with interlaced time-lapse microscopy (ITM) illumination (see Fig. 1f). Right: signal of the Lap2β nuclear membrane marker. Tracks are colored according to binding classes: long (green), intermediate (grey), and short (red). Scale bar: 5 µm.

File name: Supplementary Movie 4
Description: Example movie of HT-Rad21 binding classes in 24hpf zebrafish embryos. Left: single molecule movie of HT-Rad21 molecules recorded with interlaced time-lapse microscopy (ITM) illumination (see Fig. 1f). Right: signal of the Lap2β nuclear membrane marker. Tracks are colored according to binding classes: long (green), intermediate (grey), and short (red). Scale bar: 5 µm.

File name: Supplementary Movie 5
Description: Example movie of long-bound HT- rad21 mobility in 64-cell stage zebrafish embryos. Left: single molecule movie of HT-Rad21 molecules recorded with the long time-lapse alternated with continuous intervals (TACO) illumination (see Fig. 4a). Right: signal of the Lap2β nuclear membrane marker. Only the first continuous illumination period of tracks that meet the long detection definition are shown. Scale bar: 5 µm.

File name: Supplementary Movie 6
Description: Example movie of long-bound HT- rad21 mobility in shield stage zebrafish embryos. Left: single molecule movie of HT-Rad21 molecules recorded with the long time-lapse alternated with continuous intervals (TACO) illumination (see Fig. 4a). Right: signal of the Lap2β nuclear membrane marker. Only the first continuous illumination period of tracks that meet the long detection definition are shown. Scale bar: 5 µm.

File name: Supplementary Movie 7
Description: Example movie of short-bound HT- rad21 mobility in 64-cell stage zebrafish embryos. Left: single molecule movie of HT-Rad21 molecules recorded with the short time-lapse alternated with continuous intervals (TACO) illumination (see Fig. 4a). Right: signal of the Lap2β nuclear membrane marker. Only tracks that meet the short detection definition are shown. Scale bar: 5 µm.

File name: Supplementary Movie 8
Description: Example movie of short-bound HT- rad21 mobility in shield stage zebrafish embryos. Left: single molecule movie of HT-Rad21 molecules recorded with the short time-lapse alternated with continuous intervals (TACO) illumination (see Fig. 4a). Right: signal of the Lap2β nuclear membrane marker. Only tracks that meet the short detection definition are shown. Scale bar: 5 µm.

References

1. Pálfy, M., Schulze, G., Valen, E. & Vastenhouw, N. L. Chromatin accessibility established by Pou5f3, Sox19b and Nanog primes genes for activity during zebrafish genome activation. *PLoS Genet.* **16**, 1–25 (2020).

2. Pérez-Rico, Y. A., Barillot, E. & Shkumatava, A. Demarcation of Topologically Associating Domains Is Uncoupled from Enriched CTCF Binding in Developing Zebrafish. *iScience* **23**, 101046 (2020).

3. Kuhn, T., Hettich, J., Davtyan, R. & Gebhardt, J. C. M. Single molecule tracking and analysis framework including theory-predicted parameter settings. *Sci. Rep.* **11**, 1–12 (2021).
