# Supplementary material for: Increasingly efficient chromatin binding of cohesin and CTCF supports chromatin architecture formation during zebrafish embryogenesis
Source: Nat Commun. 2025 Feb 21;16:1833. doi: 10.1038/s41467-025-56889-5 (PMC11842872; doi:10.1038/s41467-025-56889-5)
Supplement: Supplementary file 2 — Description of Additional Supplementary Files [file 41467_2025_56889_MOESM2_ESM.pdf]

Description of Additional Supplementary Files

File name: Supplementary Movie 1
Example movie of HT-Rad21 mobility in 64-cell stage zebrafish embryo. Left: single molecule movie of HT-Rad21 molecules with continuous illumination at 11.7 ms frame cycle time (see Fig. 1c). Right: signal of the Lap2β nuclear membrane marker. Scale bar: 5 µm.

File name: Supplementary Movie 2
Example movie of HT-Rad21 mobility in shield stage zebrafish embryo**.** Left: single molecule movie of HT-Rad21 molecules with continuous illumination at 11.7 ms frame cycle time (see Fig. 1c). Right: signal of the Lap2β nuclear membrane marker. Scale bar: 5 µm.

File name: Supplementary Movie 3
Description: Example movie of HT-Rad21 binding classes in 64-cell stage zebrafish embryos. Left: single molecule movie of HT-Rad21 molecules recorded with interlaced time-lapse microscopy (ITM) illumination (see Fig. 1f). Right: signal of the Lap2β nuclear membrane marker. Tracks are colored according to binding classes: long (green), intermediate (grey), and short (red). Scale bar: 5 µm.

File name: Supplementary Movie 4
Description: Example movie of HT-Rad21 binding classes in 24hpf zebrafish embryos. Left: single molecule movie of HT-Rad21 molecules recorded with interlaced time-lapse microscopy (ITM) illumination (see Fig. 1f). Right: signal of the Lap2β nuclear membrane marker. Tracks are colored according to binding classes: long (green), intermediate (grey), and short (red). Scale bar: 5 µm.

File name: Supplementary Movie 5
Description: Example movie of long-bound HT- rad21 mobility in 64-cell stage zebrafish embryos. Left: single molecule movie of HT-Rad21 molecules recorded with the long time-lapse alternated with continuous intervals (TACO) illumination (see Fig. 4a). Right: signal of the Lap2β nuclear membrane marker. Only the first continuous illumination period of tracks that meet the long detection definition are shown. Scale bar: 5 µm.

File name: Supplementary Movie 6
Description: Example movie of long-bound HT- rad21 mobility in shield stage zebrafish embryos. Left: single molecule movie of HT-Rad21 molecules recorded with the long time-lapse alternated with continuous intervals (TACO) illumination (see Fig. 4a). Right: signal of the Lap2β nuclear membrane marker. Only the first continuous illumination period of tracks that meet the long detection definition are shown. Scale bar: 5 µm.

File name: Supplementary Movie 7
Description: Example movie of short-bound HT- rad21 mobility in 64-cell stage zebrafish embryos. Left: single molecule movie of HT-Rad21 molecules recorded with the short time-lapse alternated with continuous intervals (TACO) illumination (see Fig. 4a). Right: signal of the Lap2β nuclear membrane marker. Only tracks that meet the short detection definition are shown. Scale bar: 5 µm.

File name: Supplementary Movie 8

Description: Example movie of short-bound HT- rad21 mobility in shield stage zebrafish embryos. Left: single molecule movie of HT-Rad21 molecules recorded with the short time-lapse alternated with continuous intervals (TACO) illumination (see Fig. 4a). Right: signal of the Lap2β nuclear membrane marker. Only tracks that meet the short detection definition are shown. Scale bar: 5 µm.
