# Supplementary material for: Increasingly efficient chromatin binding of cohesin and CTCF supports chromatin architecture formation during zebrafish embryogenesis
Source: Nat Commun. 2025 Feb 21;16:1833. doi: 10.1038/s41467-025-56889-5 (PMC11842872; doi:10.1038/s41467-025-56889-5)
Supplement: Supplementary file 11 — Reporting Summary [file 41467_2025_56889_MOESM11_ESM.pdf]

nature portfolio

Corresponding author(s): J. Christof M. Gebhardt

Last updated by author(s): 01/02/2025

Reporting Summary

Nature Portfolio wishes to improve the reproducibility of the work that we publish. This form provides structure for consistency and transparency in reporting. For further information on Nature Portfolio policies, see our Editorial Policies and the Editorial Policy Checklist.

Statistics

For all statistical analyses, confirm that the following items are present in the figure legend, table legend, main text, or Methods section.

n/a	Confirmed	
☐	☒	The exact sample size (n) for each experimental group/condition, given as a discrete number and unit of measurement
☐	☒	A statement on whether measurements were taken from distinct samples or whether the same sample was measured repeatedly
☐	☒	The statistical test(s) used AND whether they are one- or two-sided<br>*Only common tests should be described solely by name; describe more complex techniques in the Methods section.*
☒	☐	A description of all covariates tested
☒	☐	A description of any assumptions or corrections, such as tests of normality and adjustment for multiple comparisons
☐	☒	A full description of the statistical parameters including central tendency (e.g. means) or other basic estimates (e.g. regression coefficient) AND variation (e.g. standard deviation) or associated estimates of uncertainty (e.g. confidence intervals)
☐	☒	For null hypothesis testing, the test statistic (e.g. F, t, r) with confidence intervals, effect sizes, degrees of freedom and P value noted<br>*Give P values as exact values whenever suitable.*
☒	☐	For Bayesian analysis, information on the choice of priors and Markov chain Monte Carlo settings
☒	☐	For hierarchical and complex designs, identification of the appropriate level for tests and full reporting of outcomes
☒	☐	Estimates of effect sizes (e.g. Cohen's d, Pearson's r), indicating how they were calculated

Our web collection on statistics for biologists contains articles on many of the points above.

Software and code

Policy information about availability of computer code

Data collection	The diameter of the injection mix droplet was analyzed using the CellSens Entry 2.3 (Build 18987) imaging software.<br>Single molecule microscopy movies were acquired on a Reflected Light-Sheet Microscope using Nikon NIS-Elements Version 4.40.00 64 bit.<br>Shield stage nuclear volume measurements were acquired on a Lattice Light Sheet Microscope using custom written software in LabVIEW (Chen et al., Science 346, 439 (2014).
Data analysis	Single-molecule tracking and analysis was performed in Matlab R2022a with the TrackIT Software and custrom scripts (Kuhn et al. Sci Rep 11, 9465 (2021), https://gitlab.com/GebhardtLab/TrackIt, https://doi.org/10.5281/zenodo.7092296).<br>Statistical tests were performed in GraphPad Prism 9.5.1.<br>Western blots were quantified in Image Lab 6.0 and lane profiles generated in Fiji (ImageJ 1.54f).<br>Nuclear volumes from shield stage measurements obtained by Lattice Light Sheet Microscopy were analyzed in Fiji (ImageJ 1.54f).

For manuscripts utilizing custom algorithms or software that are central to the research but not yet described in published literature, software must be made available to editors and reviewers. We strongly encourage code deposition in a community repository (e.g. GitHub). See the Nature Portfolio guidelines for submitting code & software for further information.

Data

Policy information about availability of data

All manuscripts must include a data availability statement. This statement should provide the following information, where applicable:

- Accession codes, unique identifiers, or web links for publicly available datasets
- A description of any restrictions on data availability
- For clinical datasets or third party data, please ensure that the statement adheres to our policy

Source data for figures are provided as a separate file named 'Source Data.xlsx'.
Single-particle tracking data and simulation data are available at Data Dryad repository and can be accessed with the following link: https://doi.org/10.5061/dryad.3bk3j9ks8
Data supporting the findings of this manuscript are additionally available from the corresponding author upon reasonable request.

Research involving human participants, their data, or biological material

Policy information about studies with human participants or human data. See also policy information about sex, gender (identity/presentation), and sexual orientation and race, ethnicity and racism.

Reporting on sex and gender	N/A
Reporting on race, ethnicity, or other socially relevant groupings	N/A
Population characteristics	N/A
Recruitment	N/A
Ethics oversight	N/A

Note that full information on the approval of the study protocol must also be provided in the manuscript.

Field-specific reporting

Please select the one below that is the best fit for your research. If you are not sure, read the appropriate sections before making your selection.

☒ Life sciences ☐ Behavioural & social sciences ☐ Ecological, evolutionary & environmental sciences

For a reference copy of the document with all sections, see nature.com/documents/nr-reporting-summary-flat.pdf

Life sciences study design

All studies must disclose on these points even when the disclosure is negative.

Sample size	For each measurement condition of single-molecule data, we measured on average 6-8 embryos on several measurement days, resulting in thousands of events in each condition (see Supplementary Tables 1, 6, 7, 9, 11, 14, 18, 20, 22, 24), sufficient to allow for a proper statistical comparison of different conditions. In our previous work (Reisser et al., Nature Communications 2018, https://doi.org/10.1038/s41467-018-07731-8), we found that a minimum of four different embryos yielded robust results.
Data exclusions	Embryos exhibiting drift due to cellular or whole embryo movements were excluded from analysis. Single nuclei with bad signal in the early stages or overall with a high labeling density due to cell-to-cell variability were excluded from analysis to prevent tracking errors.
Replication	Measurements were performed independently on a minimum of two measurement days (see Supplementary Tables 1, 6, 7, 9, 11, 14, 18, 20, 22, 24).<br>Samples for Western blots were isolated from zebrafish collected and injected on three independent days.<br>Replica measurements were performed successfully.
Randomization	On each measurement day, hundreds of embryos were collected from multiple pairs of zebrafish. For each injection condition, random embryos were selected. mRNA injection solution was prepared on every measurement day and injected independently into each embryo. Replicas were measured at different days.
Blinding	Blinding was not required in this study. We recorded and analyzed all data under the same experimental conditions to exclude any bias (see Supplementary Table 27 for Tracking parameters).

Reporting for specific materials, systems and methods

We require information from authors about some types of materials, experimental systems and methods used in many studies. Here, indicate whether each material, system or method listed is relevant to your study. If you are not sure if a list item applies to your research, read the appropriate section before selecting a response.

Materials & experimental systems

n/a	Involved in the study
☐	☒ Antibodies
☒	☐ Eukaryotic cell lines
☒	☐ Palaeontology and archaeology
☐	☒ Animals and other organisms
☒	☐ Clinical data
☒	☐ Dual use research of concern
☒	☐ Plants

Methods

n/a	Involved in the study
☒	☐ ChIP-seq
☒	☐ Flow cytometry
☒	☐ MRI-based neuroimaging

Antibodies

Antibodies used

anti-Rad21 antibody (ab992, Abcam), anti-CTCF antibody (ab128873, Abcam), anti-HA Tag antibody (2-2.2.14 #26183, Thermo Fisher Scientific), anti-Smc3 antibody (JM10-75, Thermo Fisher Scientific). Antibodies are also described in the methods section.

Validation

For anti-Rad21 and anti-CTCF we ran uninjected wild-type samples alongside samples injected with HaloTag (HT) tagged constructs to identify HT tagged protein bands in injected embryos (see Supplementary Fig. 1). For CTCF, we additionally show Western blots under uninjected wild-type conditions and ctcf-morpholino treatment (reducing CTCF levels) highlighting the endogenous CTCF band (Supplementary Figure 17) . The anti-Rad21 antibody is cited in 207 publications and the anti-CTCF antibody in 22 publications, both with verification on the manufacturer website.
anti-Rad21: https://www.abcam.com/en-us/products/primary-antibodies/rad21-antibody-ab992#
anti-CTCF: https://www.abcam.com/en-us/products/primary-antibodies/ctcf-antibody-epr7314b-chip-grade-ab128873

The anti-HA tag antibody is referenced in 195 citations with advanced verification on the manufacturers website: https://www.thermofisher.com/antibody/product/HA-Tag-Antibody-clone-2-2-2-14-Monoclonal/26183

The anti-Scm3 antibody is provided with advanced verification on the manufacturers website: https://www.thermofisher.com/antibody/product/SMC3-Antibody-clone-JM10-75-Recombinant-Monoclonal/MA5-32594

Animals and other research organisms

Policy information about studies involving animals; ARRIVE guidelines recommended for reporting animal research, and Sex and Gender in Research

Laboratory animals

Danio rerio (Zebrafish) of the Wild Indian Karyotype (WIK) aged 0.5-2 years.

Wild animals

The study did not involve wild animals.

Reporting on sex

Sex cannot be determined in zebrafish embryos at early stages used in our study.
Sex-based information was not collected.

Field-collected samples

The study did not involve samples collected from the fields.

Ethics oversight

Zebrafish were maintained following the guidelines of the EU directive 2010/63/EU, the German Animal Welfare Act and the State of Baden-Württemberg (Germany) in addition to being permitted by the Regierungspräsidium Tübingen (35/9185.46-5, 35/9185.81-5).

Note that full information on the approval of the study protocol must also be provided in the manuscript.

Plants

Seed stocks	N/A
Novel plant genotypes	N/A
Authentication	N/A
